# Multiomics analysis reveals CT83 is the most specific gene for triple negative breast cancer and its hypomethylation is oncogenic in breast cancer

**DOI:** 10.1038/s41598-021-91290-4

**Published:** 2021-06-09

**Authors:** Chen Chen, Dan Gao, Jinlong Huo, Rui Qu, Youming Guo, Xiaochi Hu, Libo Luo

**Affiliations:** Breast and Thyroid Center, The First People’s Hospital of Zunyi (The Third Affiliated Hospital of Zunyi Medical University), Fenghuang N Rd, Zunyi, 563000 Guizhou China

**Keywords:** Cancer, Breast cancer, Cancer genomics, Epigenetics, Transcriptomics

## Abstract

Triple-negative breast cancer (TNBC) is a highly aggressive breast cancer (BrC) subtype lacking effective therapeutic targets currently. The development of multi-omics databases facilities the identification of core genes for TNBC. Using TCGA-BRCA and METABRIC datasets, we identified *CT83* as the most TNBC-specific gene. By further integrating FUSCC-TNBC, CCLE, TCGA pan-cancer, Expression Atlas, and Human Protein Atlas datasets, we found *CT83* is frequently activated in TNBC and many other cancers, while it is always silenced in non-TNBC, 120 types of normal non-testis tissues, and 18 types of blood cells. Notably, according to the TCGA-BRCA methylation data, hypomethylation on chromosome X 116,463,019 to 116,463,039 is significantly correlated with the abnormal activation of *CT83* in BrC. Using Kaplan–Meier Plotter, we demonstrated that activated *CT83* is significantly associated with unfavorably overall survival in BrC and worse outcomes in some other cancers. Furthermore, GSEA suggested that the abnormal activation of *CT83* in BrC is probably oncogenic by triggering the activation of cell cycle signaling. Meanwhile, we also noticed copy number variations and mutations of *CT83* are quite rare in any cancer type, and its role in immune infiltration is not significant. In summary, we highlighted the significance of *CT83* for TNBC and presented a comprehensive bioinformatics strategy for single-gene analysis in cancer.

## Introduction

Breast cancer is heterogeneous in molecular features. Triple-negative breast cancer is one of the four classical breast cancer subtypes that account for about 15% to 20% of diagnosed breast cancer^1^. Molecularly, TNBC is characterized by the lack of estrogen receptor (*ER*), progesterone receptor (*PR*), and human epidermal growth factor receptor 2 (*HER2*)^2^. Basal breast cancer is an underlying uncertain subtype^3^ that shares an expression profile similar to that of the basal-myoepithelial layer of the normal breast and characterized by a strong expression of basal markers such as cytokeratins 5, 6, and 17^4,5^. Despite some discordance, "TNBC" and "basal breast cancer" have been used interchangeably because the majority of basal tumors are also TNBC^6^. The current clinical management of TNBC is generally challenging^7^. TNBC is more aggressive compared with other breast cancer subtypes, as patients with TNBC are usually young and more likely to suffer from worse clinical outcomes such as early relapse and visceral metastasis regardless of their higher chemosensitivity. Although the incorporation of targeted agents like poly-ADP-ribose polymerase (PARP)^8,9^ and immune checkpoint inhibitors^10,11^ in clinical practice seems promising, TNBC responses to these treatments are still vary considerably^12^. Moreover, the lack of specific targets is one of the key factors that stymieing the substantial improvement in the targeted therapy of TNBC^13^. Therefore, the identification of core molecular markers, especially TNBC-specific ones, is still critical at this moment.

Advancement in multi-omics approaches, especially RNA-seq and microarray techniques, have yielded tons of bioinformatics data, greatly driving innovation and progression of scientific studies on cancer markers. The Cancer Genome Atlas (TCGA)^14–16^ is a landmark cancer genomics program that provided genomic, epigenomic, transcriptomic, and proteomic data in over 20,000 cancer and paired normal samples spanning 33 types of cancer, including breast cancer. The Molecular Taxonomy of Breast Cancer International Consortium (METABRIC) project^17,18^ documented genomic landscapes of over 2500 breast cancer, including clinical characteristics, gene expression, copy number variations, and mutation profiles of driver genes. Fudan University Shanghai Cancer Center (FUSCC) presented comprehensive clinical, genomic, transcriptomic data of 465 primary TNBC^19^, which is the largest TNBC genomic project to date. The Cancer Cell Line Encyclopedia (CCLE)^20–22^ comprehensively characterized over 1000 types of human cancer cell lines and provided open-access bioinformatics data including but not limited to cell phenotypes, RNA-seq, DNA copy number, DNA methylation, and gene mutations. These publicly available bioinformatics data enable the systematic search for TNBC-specific molecular markers and the prediction of their biological functions.

In this study, from over 60,000 genes and 3617 breast cancer samples, we identified a TNBC-specific key gene, *CT83*, which is significantly overexpressed in TNBC but not in other breast cancer subtypes. Moreover, by analyzing multi-omic bioinformatics data in over 10,000 cancer tissues, 1739 cancer cell lines, 124 types of normal tissues, and 18 types of blood cells, we comprehensively analyzed *CT83* in breast cancer and pan-cancer, including its expression patterns (in cancer tissues, normal tissues, cancer cell lines, and blood cells), mutation profiles, copy number variations, methylation status, association with tumor-infiltrating lymphocytes, prognostic significance, and potential biological functions in breast cancer. Briefly, our data not only highlighted the importance of *CT83* for TNBC but also presented a comprehensive bioinformatics strategy for the analysis of cancer-related genes.

## Methods

### Gene screening

The TCGA-BRCA dataset downloaded from the TCGA portal^23^ (http://tcgaportal.org/download.html) and the METABRIC dataset downloaded from the cBioPortal^24,25^ (https://www.cbioportal.org/study/summary?id=brca_metabric) were used for gene screening. The integrated file in the TCGA-BRCA dataset (RNA-seq, FPKM) and the mRNA microarray data named “data_mRNA_median_Zscores.txt” in the METABRIC dataset (Microarray, Z-scores) were used for assessing gene expression levels. Data in the “PAM50” column of the TCGA-BRCA dataset and the “Pam50 + claudin-low subtype” column in the file named “data_clinical_patient.txt” of the METABRIC dataset were used for subtype determining. Data were processed with Microsoft Excel, and the t-test was calculated to evaluate statistical significance.

### Gene annotations

Gene annotations, mainly including gene synonyms, chromosome locations, transcript lengths, exon information, and protein lengths, were obtained from the Ensembl database (https://www.ensembl.org/index.html)^26,27^.

### mRNA expression

The two datasets used in the screening part and the FUSCC-TNBC dataset^19^ (RNA-seq, Log2 FPKM) downloaded from the National Omics Data Encyclopedia (NODE; https://www.biosino.org/node/project/detail/OEP000155) were used to analyze the expression of *CT83* mRNA in breast cancer tissues of different subtypes. The expression of *CT83* mRNA in cancer cell lines was analyzed with data from the Cancer Cell Line Encyclopedia (CCLE) project^20^. Specifically, the list of breast cancer cell lines was extracted from the file named “Cell_lines_annotations_20181226.txt” downloaded from the CCLE data portal (https://portals.broadinstitute.org/ccle/data), and gene expression was analyzed with the file named “CCLE_RNAseq_genes_rpkm_20180929.gct.gz” The *CT83* expression heat maps were generated using the cBioPortal^25^ (https://www.cbioportal.org) based on data from the TCGA-BRCA^14–16^ (Pan-cancer Atlas; https://www.cbioportal.org/study/summary?id=brca_tcga_pan_can_atlas_2018), METABRIC^17^ (https://www.cbioportal.org/study/summary?id=brca_metabric), and the CCLE^20^ (https://www.cbioportal.org/study/summary?id=ccle_broad_2019) dataset. RNA-seq data in “organism part” of “homo sapiens” obtained from the Expression Atlas database (https://www.ebi.ac.uk/gxa/home) were used to analyze the expression of *CT83* in normal human tissues. RNA-seq data based on TCGA and the GTEx^28^ project obtained from the GEPIA2^29^ database (http://gepia2.cancer-pku.cn) were used to investigate the expression of *CT83* in pan-cancer and paired normal tissues.

### Copy number variation (CNV)

The CNV profiles named “data_CNA.txt” and the corresponding annotation files named “meta_CNA.txt” of the TCGA-BRCA (Firehose Legacy; https://www.cbioportal.org/study/summary?id=brca_tcga), MATABRIC (https://www.cbioportal.org/study/summary?id=brca_metabric), and CCLE (https://www.cbioportal.org/study/summary?id=ccle_broad_2019) dataset obtained from the cBioPortal database were used to analyze CNV of *CT83* in breast cancer tissues and pan-cancer cell lines. The CNV profiles of *CT83* in TCGA pan-cancer tissues were obtained from the TIMER2.0^30^ database (http://timer.cistrome.org). The DNA copy number data named “data_linear_CNA.txt” and RNA-seq data named “data_RNA_Seq_v2_expression_median.txt” in the TCGA-BRCA (Firehose Legacy) were used to analyze the correlation between the *CT83* DNA copy number and mRNA expression levels. Pearson’s correlation coefficient was calculated using the GraphPad Prism (https://www.graphpad.com) to evaluate their correlation strength. R-squared was calculated to evaluate the DNA-mRNA linear correlation.

### Mutations

The TCGA-BRCA (Firehose Legacy; https://bit.ly/36wxqQP; https://bit.ly/2HRYxvb), CCLE-BRCA (https://bit.ly/36xQXjV), TCGA (Firehose Legacy; https://bit.ly/2GtsAZP; https://bit.ly/30AYhrb), and CCLE (https://bit.ly/36wxyzN; https://bit.ly/36x4wzX) dataset in cBioPortal database were used to analyze mutations of *CT83* in breast cancer tissues, breast cancer cell lines, pan-cancer tissues, and pan-cancer cell lines, respectively. The correlation analysis between the expression of *CT83* and mutations of key genes in breast cancer was performed with the TCGA-BRCA dataset in the TCGA Portal database (http://tcgaportal.org/TCGA/Breast_TCGA_BRCA/index.html). The permutation tests were auto-calculated to evaluate the correlation strength.

### Methylation

The correlation between *CT83* expression and its methylation in breast cancer tissues was analyzed based on the TCGA-BRCA dataset in the TCGA Portal database (http://tcgaportal.org/TCGA/Breast_TCGA_BRCA/index.html). The methylation status of *CT83* was shown as methylation beta-values^31^. Chromosome locations of involved methylation probes were annotated with the NCBI Genome Data Viewer (https://www.ncbi.nlm.nih.gov/genome/gdv). Pearson’s correlation coefficient was calculated using GraphPad Prism (https://www.graphpad.com) to evaluate the correlation strength^31,32^. A Pearson’s value of -0.8 or less was considered as a strong correlation.

### Immune infiltration

The TIMER2.0^30^ (http://timer.cistrome.org) and TISIDB^33^ (http://cis.hku.hk/TISIDB) databases were used to analyze the correlation between *CT83* mRNA expression and the abundance of tumor-infiltrating lymphocytes in breast cancer tissues and pan-cancer tissues, respectively. *CXorf61*, one of the aliases of *CT83*, was used for searching in both database. Spearman’s correlation coefficient was auto-calculated to evaluate the strength of correlation. An absolute Spearman’s value of 0.75 or more was considered as a strong correlation.

### Prognosis

Survival analysis was performed using the Kaplan–Meier Plotter^34^ (https://kmplot.com/analysis) with mRNA data in breast cancer (gene chips; https://kmplot.com/analysis/index.php?p=service&cancer=breast) and pan-cancer (RNA-seq; https://kmplot.com/analysis/index.php?p=service&cancer=pancancer_rnaseq). Hazard ratios (HR), 95% confidence intervals (95%CI), and log-rank test* P*-values were all auto-calculated by the Kaplan–Meier Plotter. *CT83* will be considered as prognostic if a *P*-value is 0.05 or less.

### Gene set enrichment analysis

Gene set enrichment analysis (GSEA)^35^ was performed according to the official guidelines (https://software.broadinstitute.org/cancer/software/gsea/wiki/index.php/Main_Page). Briefly, the TCGA-BRCA (http://tcgaportal.org/download.html) and METABRIC (https://www.cbioportal.org/study/summary?id=brca_metabric) datasets were downloaded to get gene expression profiles in breast cancer tissues. Next, the “expression dataset” and the “phenotype labels” were prepared based on the required format (https://www.gsea-msigdb.org/gsea/doc/GSEAUserGuideFrame.html?Preparing_Data_Files). Annotated datasets were then analyzed with the GSEA software (v4.1.0, https://www.gsea-msigdb.org/gsea/downloads.jsp) using the the hallmark gene sets (h.all.v7.2.symbols.gmt) and the KEGG gene sets (c2.cp.kegg.v7.2.symbols.gmt). Samples were divided into *CT83*-positive and *CT83*-negative cohorts using 1 FPKM (for TCGA-BRCA) and 1 Z-score (for METABRIC) as the cutoffs, respectively. The top 5 positively and negatively enriched gene sets were included for further analysis. Enriched genes that were annotated as “core enrichment” in the Cell Cycle, G2M Checkpoint, and E2F targets were used for the screening of *CT83*-associated core genes. The expression profiles of *CT83* and its correlated genes in breast cancer were obtained using the TCGA-BRCA, METABRIC, and the CCLE-BRCA dataset in the cBioPortal database (https://www.cbioportal.org).

## Results

### CT83 is significantly overexpressed in TNBC but not in other breast cancer subtypes

**Figure 1:**
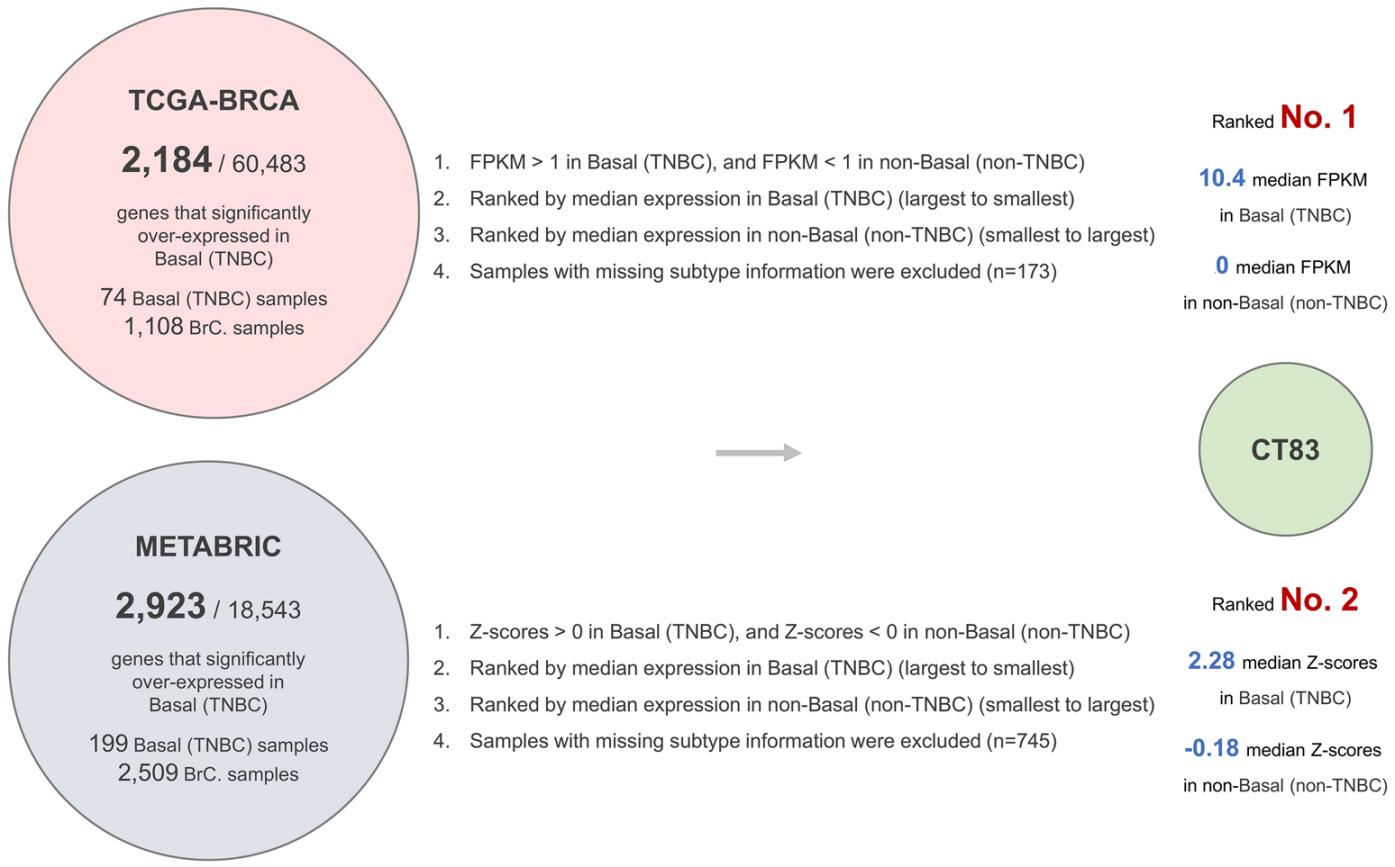
Screening strategies and basic annotations of *CT83*. Screening strategies for genes that significantly overexpressed in TNBC but not in non-TNBC subtypes. (**B**) The location of *CT83* on chromosome X. (**C**) The schematic diagram of the only transcript of *CT83*.

To screen for genes that significantly overexpressed in TNBC but not in other breast cancer subtypes, we analyzed the RNA-seq data from the TCGA-BRCA and the mRNA microarray data from the METABRIC datasets. Based on our screening criteria, *CT83* is the top-ranked gene in the TCGA-BRCA dataset and second-ranked in the METABRIC dataset (Fig. 1A; Table S1). The median expression of *CT83* in TNBC tissues is 10.4 FPKM in the TCGA-BRCA dataset and 2.28 Z-score in the METABRIC dataset, while its maximal median expression levels in non-TNBC tissues are 0 FPKM and -0.18 Z-score, respectively. Cancer/testis antigen 83 (*CT83*), also known as *CXorf61*, *FLJ20611*, *FLJ22913*, and *KK-LC-1*, is a protein-coding gene located on the reverse strand of chromosome X (Fig. 1B). It has only one transcript with a length of 517 bp that consisted of 2 exons (Fig. 1C). The protein encoded by *CT83* mRNA is 113 aa in length. As the TNBC-specific expression patterns of *CT83* were observed in two independent datasets with large sample sizes, we hypothesized that *CT83* may play distinct roles in TNBC.

### CT83 is frequently overexpressed in TNBC tissues and cell lines

**Figure 2:**
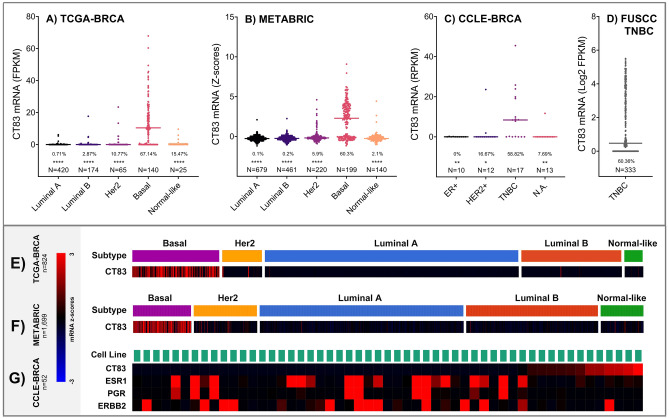
The expression of *CT83* mRNA in breast cancer. The expression of *CT83* mRNA in breast cancer tissues of different subtypes according to data from the TCGA-BRCA (**A**,**E**) and METABRIC (**B**,**F**) dataset. The expression of *CT83* mRNA in TNBC tissues according to data from the FUSCC-TNBC dataset (**D**). The expression of *CT83* mRNA in breast cancer cell lines based on data from the CCLE-BRCA dataset (**C**,**G**). The expression of *ESR1*, *PGR,* and *ERBB2* was plotted for distinguishing the subtype of breast cancer cell lines. The median expression of *CT83* in each subtype was labeled as dashes. The positive rate of *CT83* mRNA was labeled as percentage numbers in A (> 1 FPKM), 2C (> 1 RPKM), and 2D (> 1 Log2 FPKM), while the positive rate of *CT83* in B was not presented because the positive expression cutoff cannot be determined based on the METABRIC raw data. Only samples with available both *CT83* expression and subtype information were used for plotting. The asterisks in A–C represent the statistical difference (t-test *p* values) of *CT83* expression between the Basal subtype and other subtypes. *, *p* < 0.05; **, *p* < 0.01; ***, *p* < 0.001; ****, *p* < 0.0001.

Next, we investigated the RNA-seq and microarray data from the TCGA-BRCA, METABRIC, FUSCC-TNBC, and CCLE-BRCA dataset to get a better understanding of the expression *CT83* mRNA in breast cancer tissues and cell lines (Fig. 2). As mentioned in the screening part, the expression of *CT83* mRNA is significantly higher in Basal/TNBC tissues compared with other subtypes, and a similar trend was observed in the 57 breast cancer cell lines (including 17 TNBC cell lines) in which the expression of *CT83* mRNA was measured (Table S2). Moreover, the positive rate of *CT83* mRNA is the highest in either TNBC tissues or cell lines, while its expression is always undetectable in breast cancer tissues or cell lines of other subtypes. The frequency of detectable *CT83* mRNA, defined as 1 FPKM/RPKM/Z-score or more, was 67.1% (94/140), 60.3% (125/199), 60.4% (201/333), and 58.8% (10/17) according to data from the TCGA-BRCA, METABRIC, FUSCC-TNBC, and CCLE-BRCA-TNBC dataset, respectively. Although the expression data of *CT83* protein was not available in any of the above-mentioned dataset or the Clinical Proteomic Tumor Analysis Consortium (CPTAC) dataset^36^, data from a previous study suggested that the protein of *CT83* is always detectable in TNBC tissues and cell lines^37^. Briefly, data from 672 TNBC tissues, 2184 non-TNBC breast cancer tissues, 17 TNBC cell lines, and 35 non-TNBC breast cancer cell lines demonstrated that *CT83* is distinctively and frequently overexpressed in TNBC.

### CT83 expression is highly restricted in normal tissues but widely distributed in cancer tissues and cell lines

**Figure 3:**
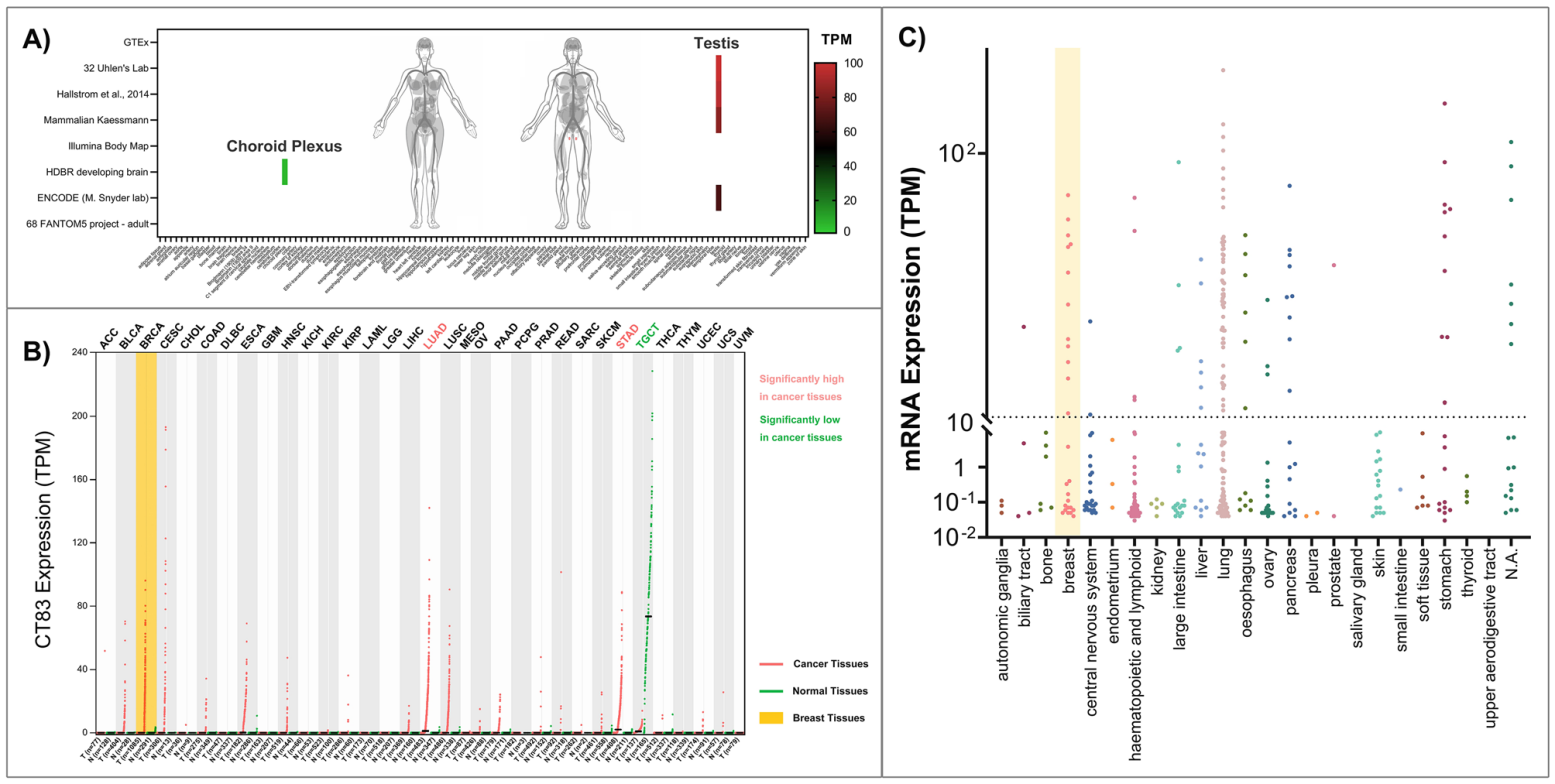
The expression of *CT83* in normal tissues, cancer tissues, and cancer cell lines. (**A**) The expression of *CT83* in normal adult tissues based on 8 RNA-seq datasets obtained from the Expression Atlas. (**B**) The expression of *CT83* in pan-cancer and paired normal tissues based on RNA-seq data from the GEPIA2 database. The abbreviations of involved cancers are available on this webpage (https://gdc.cancer.gov/resources-tcga-users/tcga-code-tables/tcga-study-abbreviations). (**C**) The expression of *CT83* in pan-cancer cell lines based on RNA-seq data from the CCLE dataset.

Since the expression of cancer/testis antigens is highly restricted in normal adult tissues and widely distributed in tumor tissues^38,39^, we examined the expression of *CT83* in 120 types of normal human tissues (Fig. 3A), 33 types of cancer tissues (Fig. 3B), 1019 cancer cell lines (Fig. 3C), and 18 types of blood cells (Figure S1). Based on RNA-seq data of 8 datasets in the Expression Atlas database, the expression of *CT83* can only be detected in testis tissues in normal adult males and sometimes in brain (choroid plexus) tissues, while no positive *CT83* expression is observed in any other normal tissue. Conversely, the abnormal activation of *CT83* is often detected in cancer tissues including breast cancer, and its high expression is statistically different from paired normal tissues in lung adenocarcinoma (LUAD) and stomach adenocarcinoma (STAD) tissues. Moreover, the high expression of *CT83* mRNA, defined as 10 TPM or more, is also often detected in cancer cell lines, especially in cell lines of breast cancer, liver cancer, lung cancer, pancreas cancer, and stomach cancer. These expression patterns of *CT83* are consistent with previous understanding about typical cancer/testis antigens, indicating that *CT83* is a potential therapeutic target in cancer immunotherapy^40^.

### Copy number variation is not the major cause of the abnormal activation of CT83 in cancer

**Figure 4:**
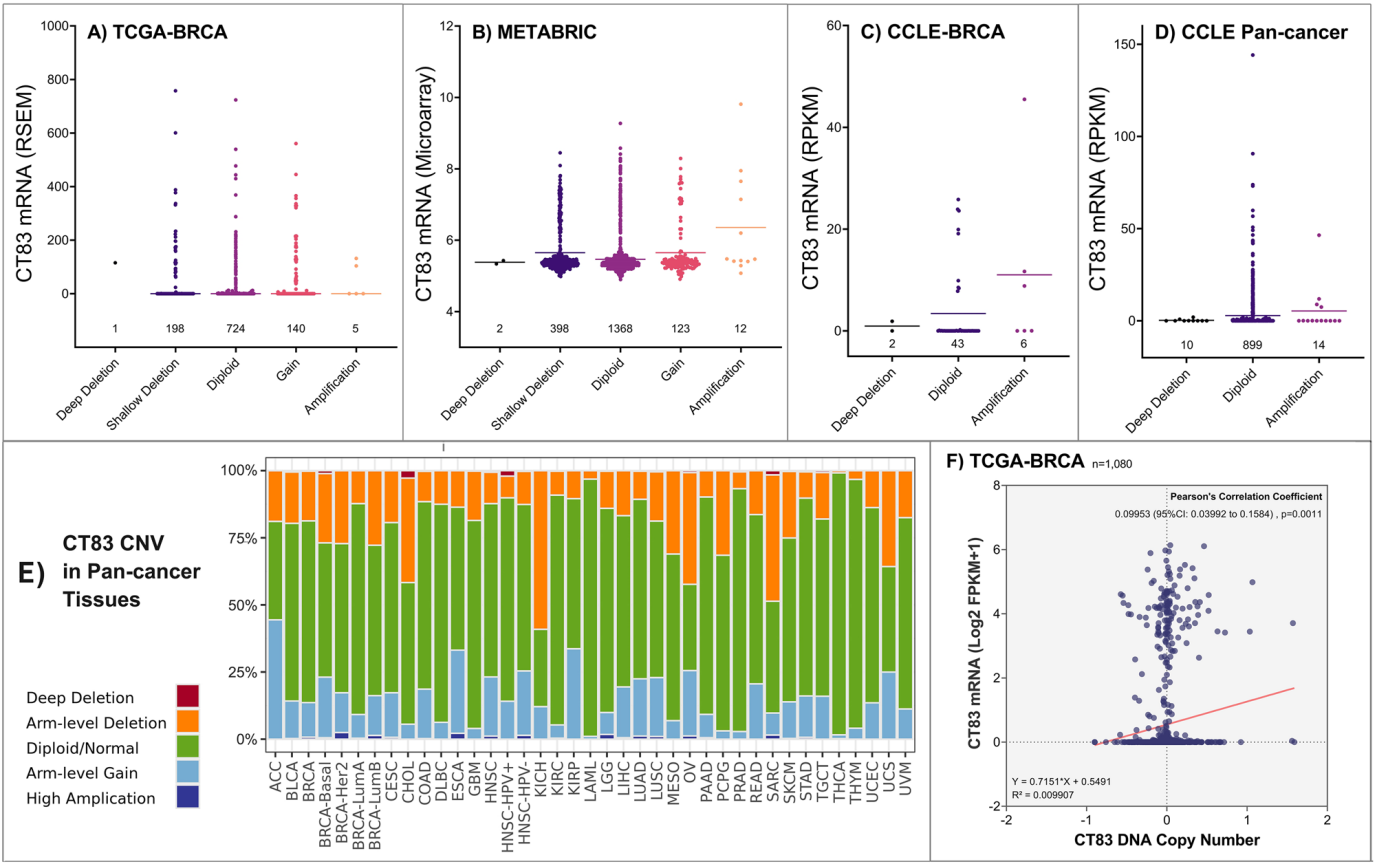
Copy number variations of *CT83* in cancer. Copy number variations of *CT83* in breast cancer tissues based on data from the TCGA-BRCA (**A**) and the METABRIC (**B**) dataset. Copy number variations of *CT83* in breast cancer cell lines (**C**) and pan-cancer cell lines (**D**) based on data from the CCLE dataset. The median expression levels of *CT83* mRNA were labeled as dashes. Copy number variations of *CT83* in pan-cancer tissues (**E**) based on TCGA cancer data. The correlation between *CT83* DNA copy number and *CT83* mRNA expression levels (**F**).

Copy number variation (CNV), especially copy number amplification, is one of the major causes of gene overexpression in cancer^41–43^. Therefore, we investigated the correlation between the expression level of *CT83* mRNA and the copy number status of its DNA to figure out whether the abnormal activation of *CT83* in cancer is a consequence of its copy number amplification. According to data from the TCGA-BRCA and the METABRIC dataset, copy number variation (amplification and deep deletion) of *CT83* is a rare event in breast cancer tissues (Fig. 4A,B). Among the included 2971 breast cancer tissue samples, *CT83* amplification rate was only less than 1% in both datasets. Similarly, *CT83* amplification was detected in 6/51 of the CCLE-BRCA cell lines, while its deep deletion was observed in only 2 breast cancer cell lines (Fig. 4C). Apart from breast cancer, copy number variation of *CT83* is also uncommon in pan-cancer tissues and cell lines (Fig. 4D,E). Specifically, *CT83* amplification was detected in 14/923 (1.52%) cancer cell lines based on CCLE pan-cancer CNV data. In 33 types of TCGA cancer tissues, most of the detected *CT83* DNA were diploid, while its DNA amplification or deep deletion was still rare events. Moreover, we also analyzed the linear correlation between its DNA copy number and mRNA expression levels with data from the TCGA-BRCA dataset (Fig. 4F). The R-squared and Pearson’s correlation coefficients of their correlation was 0.009907 and 0.09953 (*p* = 0.0011), respectively. Taken together, these data showed that copy number variation has no significant correlation with the abnormal activation of *CT83* in breast cancer and in other cancers.

### CT83 mutation is a rare event in cancer

**Figure 5:**
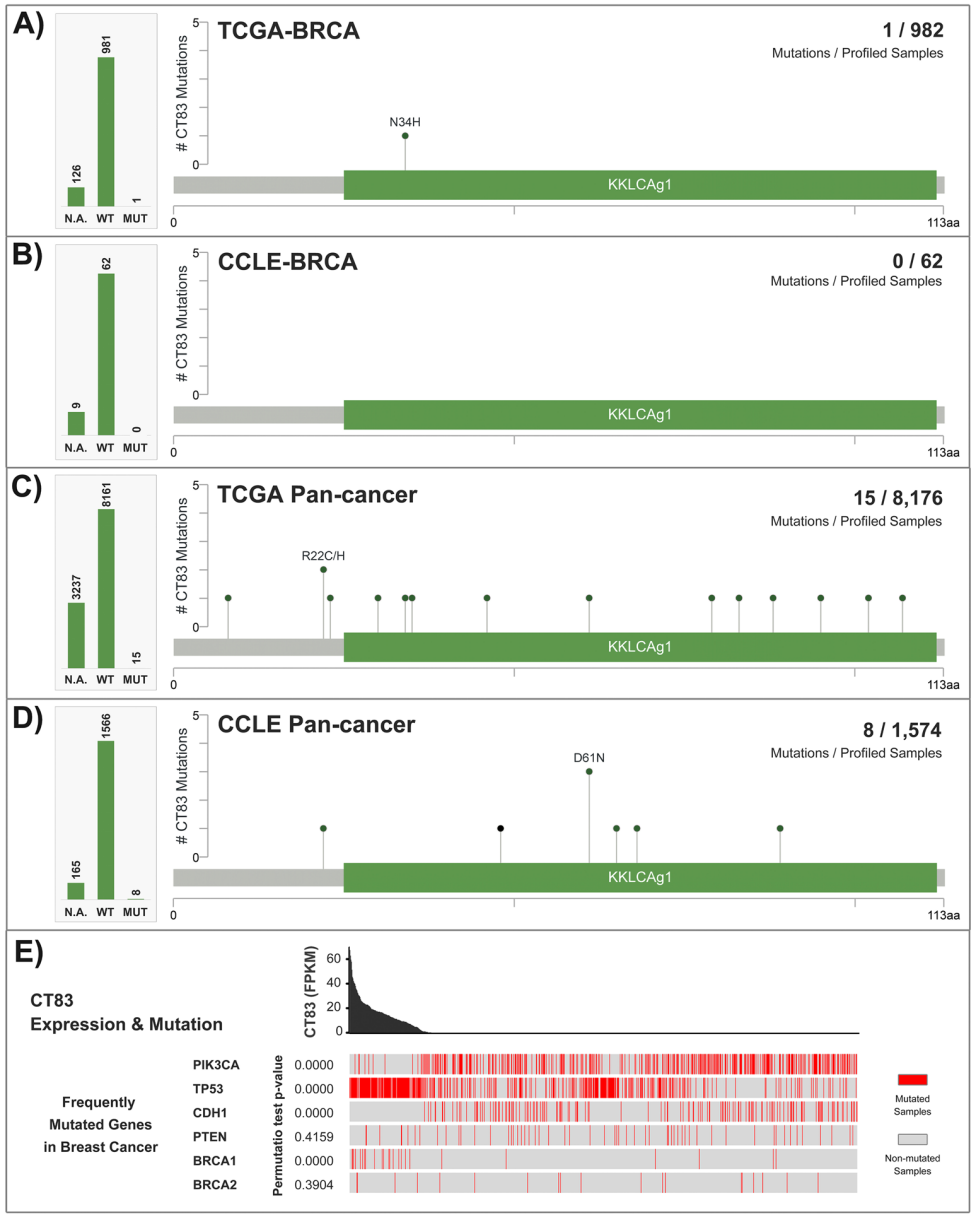
Mutations of *CT83* in cancer. (**A**) *CT83* mutations in breast cancer tissues, (**B**) breast cancer cell lines, (**C**) TCGA pan-cancer tissues, and (**D**) CCLE pan-cancer cell lines. (**E**) The correlation between *CT83* mRNA expression and mutations of key genes in breast cancer based on data from the TCGA-BRCA dataset. *CT83* mutation points were labeled in the corresponding regions in the linear schematic diagram of its protein. Permutation test *p*-values were calculated by comparing *CT83* expression in key-gene-mutated samples with key-gene-unmutated samples. N.A., not available; WT, wild type; MUT, mutated.

Somatic mutations also contribute to changes in gene expression^44^, and some driver mutations are critical for cancer development^45,46^. Hence, we examined mutation profiles of *CT83* in cancer. In 982 TCGA-BRCA samples with available *CT83* mutation data, only one *CT83* mutation was detected, while not any *CT83* mutation was observed in the 62 CCLE-BRCA cell lines (Fig. 5A,B). In 8176 TCGA pan-cancer tissues spanning 33 cancer types, mutated *CT83* was only detected in 15 samples (Fig. 5C). Similarly, among the 1574 CCLE pan-cancer cell lines, *CT83* mutations were only observed in 8 cell lines of 7 mixed cancer types (Fig. 5D). Moreover, *CT83* mutations were sporadic that no mutation hotspot was identified (Table S3). In addition, we also assessed the correlation between *CT83* mRNA expression levels and mutations of key genes in breast cancer (Fig. 5E). According to the TCGA-BRCA dataset, *CT83* expression is statistically higher in TP53-mut (permutation test *p* = 0.0000) or BRCA1-mut samples (permutation test *p* = 0.0000), significantly lower in PIK3CA-mut (permutation test *p* = 0.0000) or CDH1-mut samples (permutation test *p* = 0.0000), and has no significant correlation with PTEN and BRCA2 mutations. Briefly, data from this part suggested that mutation is not one of the major genomic alternations of *CT83* in cancer.

### Hypomethylation may induce the abnormal activation of CT83 in cancer

**Figure 6:**
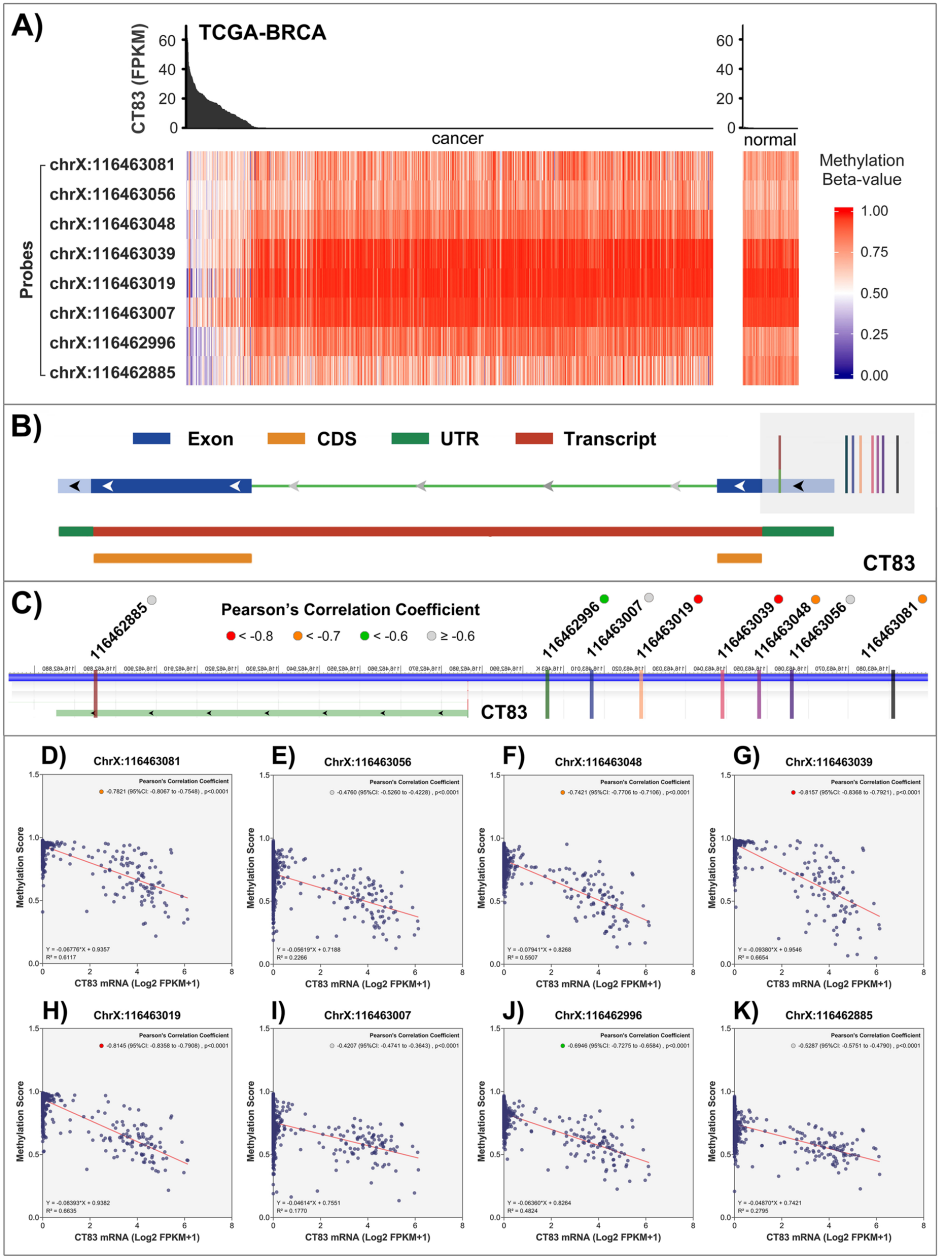
*CT83* methylation in breast cancer. (**A**) The correlation between *CT83* mRNA expression and its methylation status in breast cancer tissues. (**B**) Locations of *CT83* methylation probes relative to *CT83* DNA sequence. It should be noted that *CT83* is located on the reverse strand of chromosome X. (**C**) Enlarged views for locations *CT83* methylation probes. Pearson’s correlation coefficients between *CT83* mRNA expression levels and methylation beta values were labeled as colorful dots. D-K) Pearson’s correlation coefficients between *CT83* mRNA expression levels and methylation beta values of corresponding probes.

Previous studies have identified that epigenetic modification, especially hypomethylation, is of great importance in the abnormal activation of CT genes in cancer^47–49^. Therefore, we analyzed the correlation between *CT83* mRNA expression levels and its methylation status in breast cancer tissues with the TCGA-BRCA dataset (Fig. 6A). The expression level of *CT83* is usually higher when some of its DNA loci are hypomethylated, while *CT83* is always silenced when its DNA is hypermethylated. As 8 probes were used to measure *CT83* methylation status at different DNA loci, we then assessed these data in detail to find out the DNA locus in which its methylation status is mostly responsible for the expression changes of *CT83* (Fig. 6B,C). By evaluating the correlation between the methylation score of a specific probe and the expression level of *CT83* mRNA (Fig. 6D–K), we found that methylation of the region between 116,463,019 to 116,463,039 on chromosome X is most closely correlated with *CT83* expression. The Pearson’s correlation coefficients for the probe “ChrX:116,463,039” (Fig. 6G) and “ChrX:116,463,019” (Fig. 6H) are − 0.8157 (95% CI − 0.8368 to − 0.7921, *p* < 0.0001) and − 0.8145 (95% CI − 0.8358 to − 0.7908, *p* < 0.0001), respectively. These data indicated that hypomethylation of the region 116,463,019 to 116,463,039 on chromosome X may induce the abnormal activation of *CT83* in breast cancer tissues.

### CT83 and tumor-infiltrating lymphocytes

The association between cancer/testis antigens and immune components has been widely reported^50–52^. We analyzed the TIMER2.0 and TISIDB database to assess the correlation between the expression of *CT83* and the abundance of tumor-infiltrating lymphocytes (TILs). According to data from the TIMER2.0 database, the expression of *CT83* has no significant correlation with any types of TILs in breast cancer tissues or any breast cancer subtypes, with the strongest correlation observed in “T cell CD4 + Th2_XCELL” of the overall cohort (Spearman’s correlation coefficient = 0.4213, *p* = 0.0000) (Table S4). Moreover, based on data from the TISIDB database, the abundance of TILs in pan-cancer tissues is independent of *CT83* expression (Figure S2). For the strongest correlation, *CT83* mRNA expression is positively correlated with the abundance of type 17 T helper cells (Th17) in esophageal carcinoma (ESCA) (Spearman’s correlation coefficient = 0.442, *p* = 0.0000) and negatively correlated with effector memory CD4 T cells (Tem_CD4) in rectum adenocarcinoma (READ) (Spearman’s correlation coefficient = − 0.413, *p* = 0.0000).

### Prognostic analysis

**Figure 7:**
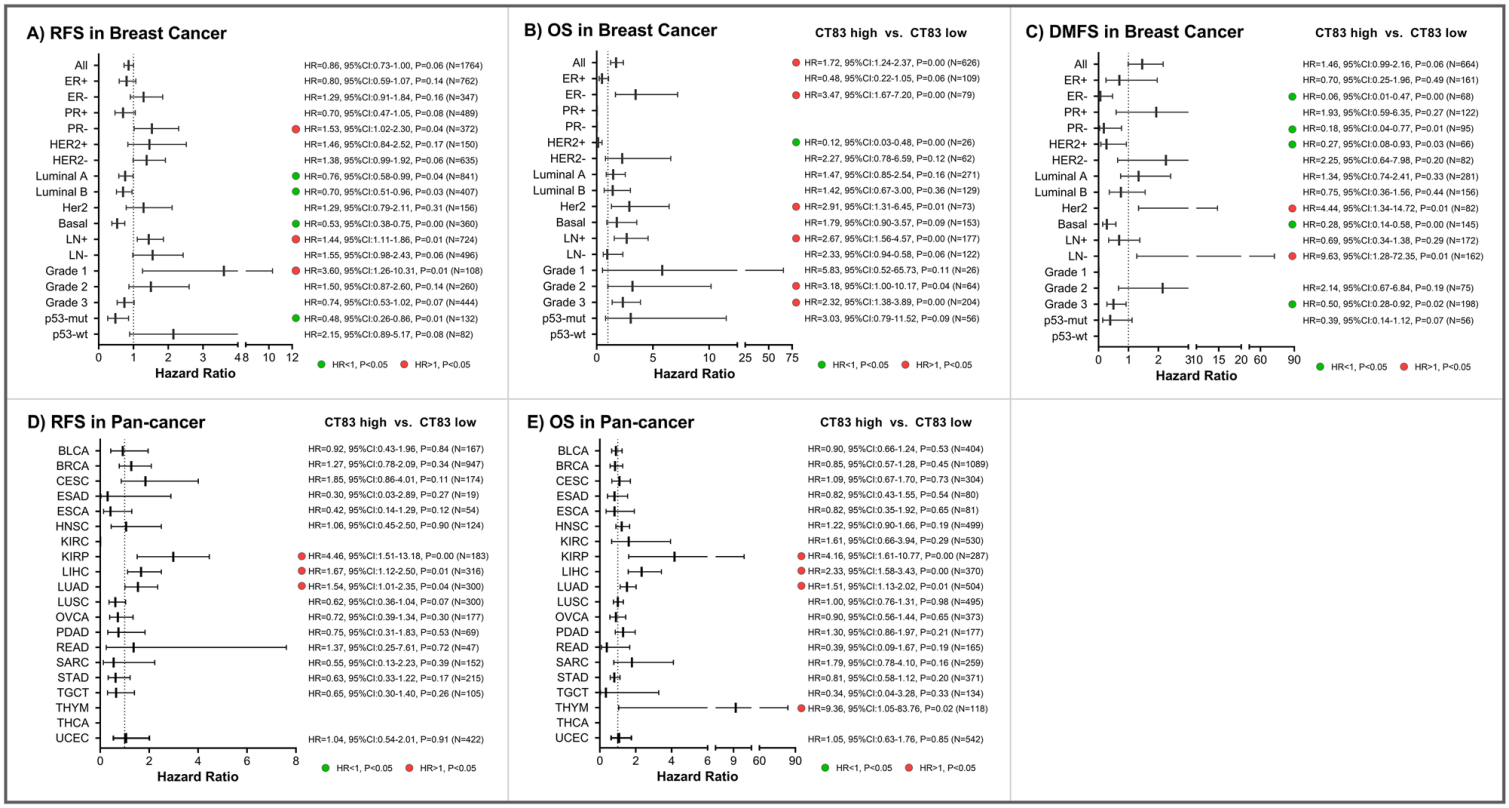
Prognostic significance of *CT83* in breast cancer and pan-cancer. The hazard ratios of *CT83* (high vs. low) for (**A**) RFS, (**B**) OS, and (**C**) DMFS in breast cancer grouped by different clinical characteristics. The hazard ratios of *CT83* (high vs. low) for (**D**) RFS, (**E**) OS in different cancers. Abbreviations: RFS, relapse-free survival; OS, overall survival; DMFS, distant metastases-free survival; HR, hazard ratio; 95%CI, 95% confidence interval; P, log-rank test* P*-value; see https://gdc.cancer.gov/resources-tcga-users/tcga-code-tables/tcga-study-abbreviations for TCGA study abbreviations.

Expression of cancer/testis antigens in cancer is frequently associated with prognosis, both unfavorable prognosis and improved outcomes^53–57^. Based on this, we assessed the prognostic significance of *CT83* in breast cancer as well as in pan-cancer using the KM Plotter database. In breast cancer (Fig. 7A–C), high *CT83* expression is statistically associated with poor OS (overall survival; HR = 1.72, 95% CI 1.24–2.37, *p* = 0.00, N = 626) but not with RFS (relapse-free survival; HR = 0.86, 95% CI 0.73–1.00, *p* = 0.06, N = 1764) and DMFS (distant metastasis-free survival; HR = 1.46, 95% CI: 0.99–2.16, *p* = 0.06, N = 664) in the overall analysis. In breast cancer subgroup analysis, high *CT83* is considered as an unfavorable risk factor in PR-negative, node-positive, and Grade-1 subgroups for RFS, in ER-negative, HER2 subtype, node-positive, Grade-2, and Grade-3 subgroups for OS, and in HER2 subtype and node-negative subgroups for DMFS. Meanwhile, *CT83* high expression is also associated with improved outcomes for RFS in Luminal A, Luminal B, Basal, and p53-mut subgroups, for OS in the HER2-positive subgroup, and for DMFS in ER-negative, PR-negative, HER2-positive, Basal subtype, and Grade-3 subgroups. In pan-cancer analysis (Fig. 7D,E), *CT83* high expression is correlated with worse RFS in kidney renal papillary cell carcinoma (KIRP), liver hepatocellular carcinoma (LIHC), and lung adenocarcinoma (LUAD). Similarly, in KIRP, LIHC, LUAD, and Thymoma (THYM), *CT83* high expression is associated with shorter OS. In brief, high *CT83* mRNA expression is usually unfavorably prognostic for OS in breast cancer, and correlated with worse RFS and OS in KIRP, LIHC, LUAD, and THYM.

### Gene set enrichment analysis of CT83 in breast cancer

**Figure 8:**
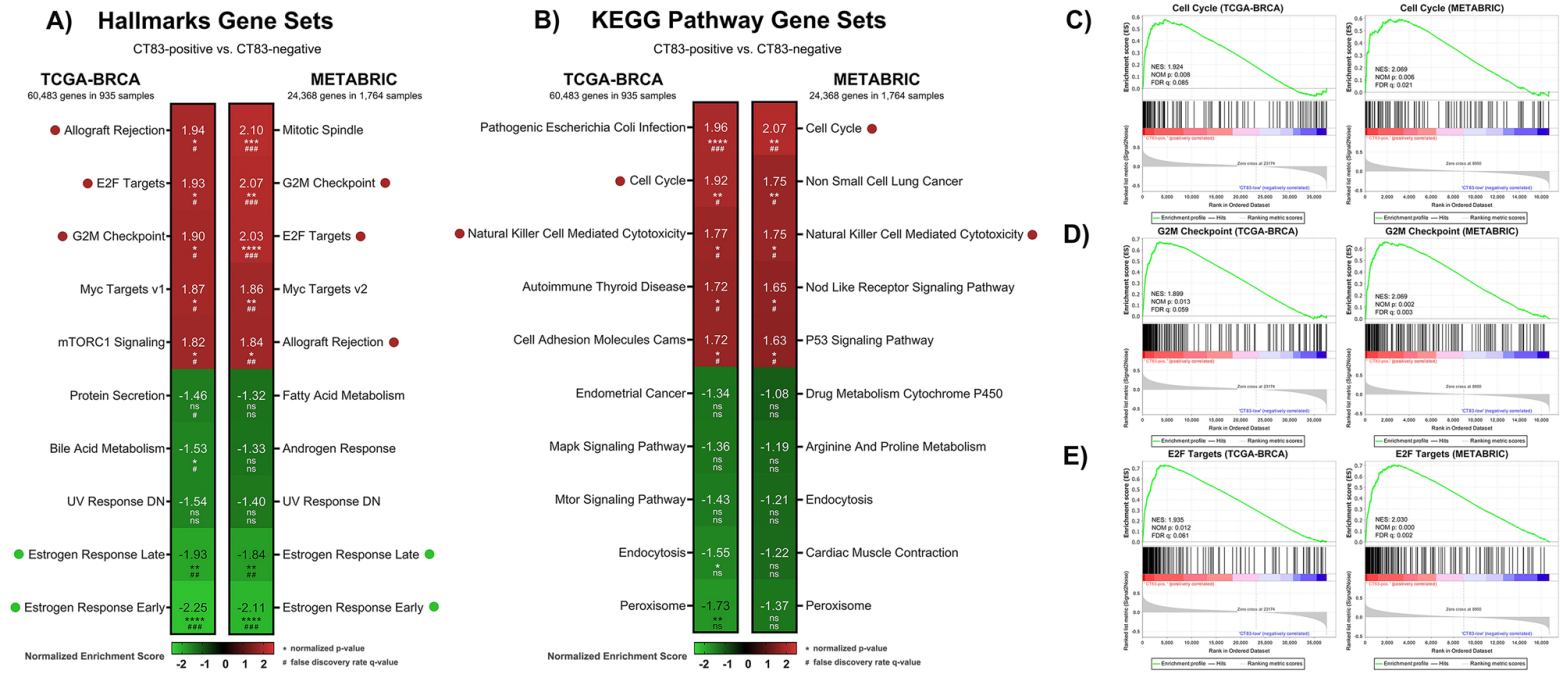
Bioinformatics prediction of *CT83* biological functions in breast cancer by GSEA. GSEA of *CT83* in breast cancer for (**A**) hallmark gene sets and (**B**) KEGG pathway gene sets using the TCGA-BRCA and METABRIC dataset. The red dots represent gene sets positively correlated with *CT83* that are common in the TCGA-BRCA and METABRIC dataset with statistical significance, while the green dots were negatively correlated shared gene sets. Details of the three enriched gene sets associated with cell cycle signaling, namely (**C**) Cell Cycle, (**D**) G2M Checkpoint, and (**E**) E2F targets. Abbreviations: NES, normalized enrichment score; NOM p, normalized* P*-value; FDR q, false discovery rate q-value; *, *p* < 0.05; **, *p* < 0.01; ***, *p* < 0.001; ****, *p* < 0.0001; ^#, ^q < 0.25; ^##, ^q < 0.05; ^###, ^q < 0.01; ^####, ^q < 0.001.

The biological role of *CT83* in breast cancer is currently poorly understood. Hence, we performed gene set enrichment analysis (GSEA)^35^ with the TCGA-BRCA and METABRIC dataset to predict potential functions of *CT83* in breast cancer (Fig. 8A,B). By dividing samples into *CT83*-positive and *CT83*-negative cohorts, we found that positive *CT83* expression is usually correlated with the activation of the Allograft Rejection, E2F Targets, G2M Checkpoint, Cell Cycle, and Natural Killer Cell Mediated Cytotoxicity pathways. Meanwhile, high *CT83* expression is also negatively related to the activation of the Estrogen Response Late and Estrogen Response Early pathway. Since deregulation of cell cycle signaling is one of the hallmarks of breast cancer^58,59^, we then focused on the three cell-cycle-related gene sets that were commonly enriched in both TCGA-BRCA and METABRIC, namely the Cell Cycle, G2M Checkpoint, and E2F Targets pathway (Fig. 8C–E). Based on the enrichment scores of GSEA results, we screened for core enriched genes that were shared in the three gene sets (Figure S3). A total of 16 shared core enriched genes were identified, including *CCNB2, CDC20, CDC25A, CDC25B, CDK1, CHEK1, ESPL1, MAD2L1, MCM2, MCM3, MCM5, MCM6, MYC, ORC6, PLK1*, and *PTTG1*. These data suggested that the positive expression of *CT83* in breast cancer is correlated with the activation of cell cycle signaling, especially the activation of the 16 shared core enriched genes in Cell Cycle, G2M Checkpoint, and E2F Targets pathways.

Next, we examined the expression of the 16 shared core enriched genes in breast cancer using the TCGA-BRCA, METABRIC, and CCLE-BRCA datasets (Figure S4). Compared with other subtypes, expression levels of the 16 genes were usually up-regulated in TNBC samples (Figure S5). In other words, the overexpression of the 16 genes is more frequently observed in *CT83*-positive breast cancer, while their expression levels are generally low in *CT83*-negative breast cancer. Furthermore, overexpression of the 16 genes is usually oncogenic by interacting cell cycle signals^60–64^. Taken together, these data indicated that the aberrant activation of *CT83* may interact with one or more of the 16 cell cycle correlated genes to promote tumorigenesis in breast cancer.

## Discussion

Cancer/testis (CT) antigens generally refer to a group of proteins with restricted expression in male germ cells in the testis but silenced in other somatic tissues in normal adults^38^. Extensive data have shown that the abnormal activation of CTAs is frequently observed in cancer tissues^65,66^, which is one of the driving forces of tumorigenesis^67^. Currently, more than 200 cancer-related CTAs have been discovered^68^, and most of them were well documented in the CTDatabase (http://www.cta.lncc.br). Most of CTAs are encoded by genes on chromosome X and thus be classified as CT-X antigens, while those on autosomes are defined as non-X CTAs^68^. Typically, the expression of CT-X antigens is more testis-restricted than non-X CTAs^38^. CTAs are of great importance in cancer, especially for cancer immunotherapy^65,69^. Firstly, CTAs are tumor-specific as their expression is highly restricted in normal tissues and widely distributed in cancer cells^65^. Secondly, CTAs are immunogenic partly because of the immune-privileged properties of the testis, which can be utilized for the design of anti-cancer vaccines^66,70^. For example, *NY-ESO-1*, one of the immunogenic CTAs, can significantly trigger spontaneous, humoral, and cell-mediated immune responses in cancer patients, as can be proved by the presence of serum anti-NY-ESO-1 antibody^71^ and NY-ESO-1-specific CD8+ T cells^69^. Moreover, owing to the remarkable resemblances shared by the development of cancer cells and germ cells^67^, CTAs are considered as markers for cancer stem cells (CSCs)^68^, and the aberrant expression of CTAs is also associated with the cancer transformation and abnormal differentiation of CSCs^72^. Lastly, despite the biological functions of the majority of CTAs remain unknown^68,73^, emerging evidence has highlighted their crucial roles in cellular processes, including cell cycle regulation, cell survival, apoptosis, and signal transduction^68,74^.

*CT83*, one of the CT-X antigens, is poorly understood at the current stage. Searching Pubmed with keywords “*CT83*” and its commonly used synonyms like “*CXorf61*” and “*KK-LC-1*,” there were only about 20 studies available regarding *CT83* in cancer, mostly in breast cancer, lung cancer, gastric cancer, and hepatocellular cancer. *CT83* was first identified as a tumor-specific antigen in a study on lung cancer in 2006, and was then designated as Kita-kyushu lung cancer antigen 1 (*KK-LC-1*)^75^. In lung cancer, *CT83* is one of the most frequently activated CTAs that its overexpression was often detected in lung cancer tissues and cell lines^76–78^. Moreover, the abnormal activation of *CT83* is associated with advanced tumor stages and unfavorable prognosis in patients with lung cancer^77,79, 80^. In gastric cancer, *CT83* is also frequently overexpressed and related to poor outcomes^81,82^. Interestingly, the aberrant expression of *CT83* in the stomach is associated with Helicobacter pylori infection and atrophic status, and its expression in precancerous gastric lesions was considered as a potential predictor of gastric cancer^83–85^. Regarding *CT83* in breast cancer, several studies have identified and emphasized its TNBC specificity but with a limited sample size^37,86,87^. Compared with previous findings, our data provided much stronger evidence to support this by analyzing its expression in 3617 breast cancer tissues and 71 breast cancer cell lines. In addition, *CT83* was generally considered as an ideal target for immunotherapy in breast cancer^40,88^ and lung cancer^89,90^, especially for T-cell-based targeted immunotherapy. Furthermore, there is no risk of intra-family cross-reactivity as *CT83* is the only member of its family^90^. However, apart from its expression patterns, prognostic values, and its significance for immunotherapy, very little is known about the biological functions of *CT83*, with only one paper available in Pubmed. Chen et al. reported that the abnormal activation of *CT83* could promote tumor progression through activating the Notch1/Hes1 signaling in hepatocellular carcinoma^91^.

There are several web-based platforms that provide comprehensive strategies for gene analysis in cancer, such as the cBioPortal^25^. Nevertheless, certain limitations impede the use of a single platform for a highly customized analysis. For example, gene expression data in normal tissues and normal blood cells cannot be obtained from the cBioPortal, but can be easily retrieved from the GEPIA2 and the HPA database, respectively. Moreover, the KM Plotter provided more powerful tools to perform survival analysis, as the sample size in KM Plotter is larger and subgroup analysis is ready-to-use. Similarly, the formatted methylation data in the TCGA Portal is more understandable than the raw TCGA data. Therefore, we presented a comprehensive bioinformatics strategy for the analysis of cancer-related genes, including their expression patterns, genetic alternations, epigenetic modifications, correlation with TILS, prognostic significance, and potential biological functions. Briefly, there are several basic criteria of our strategies: (1) included datasets or platforms should be publicly available; (2) raw data should be easily retrieved if possible; (3) analysis could be performed with code-free methods; (4) the whole workflow should be customizable based on specific purposes. We believe these criteria guarantee the comprehensibility and the repeatability of the associated methods and results.

The reliability of our strategies can be supported by many previous laboratory-confirmed findings, as described in the above review of *CT83*. Moreover, some of our bioinformatics findings will probably facilitate further laboratory studies on *CT83*. For example, based on the CCLE-BRCA dataset, the majority of breast cancer cell lines were *CT83*-negative, even some TNBC cell lines (such as MDA-MB-453, HCC1187, and DU4475), which is quite helpful by guiding our choice of appropriate cell models. Similarly, data from TCGA-BRCA suggested that CNVs and mutations of *CT83* in breast cancer or pan-cancer are quite rare, thus the two areas should not be the research priority in future studies. Meanwhile, we predicted that the hypomethylation of the region 116,463,019 to 116,463,039 on chromosome X may induce the abnormal activation of *CT83* in breast cancer. Several independent studies support the reliability of this prediction. Firstly, the expression of many CTAs is regulated through epigenetic modifications especially DNA methylation, as strong correlations were observed between the methylation levels of their promoters and the expression levels of CTAs in cancer^48^. Secondly, hypomethylation of *CT83* was often detected in *CT83*-positive breast cancer samples^40^, and the hypomethylating agent 5-aza-2′-deoxycytidine can active *CT83* expression in *CT83*-negative cells^37^. However, data regarding the exact locations of DNA methylation that responsible for the abnormal activation of *CT83* is not available in previous publications. We will present further evidence on this in our future studies.

Apart from *CT83*, the other TNBC-specific genes presented in table S1 may also play critical roles in TNBC, such as *HORMAD1* and *ART3*. For example, *HORMAD1* overexpression is associated with homologous recombination deficiency in triple-negative breast cancers^92^. Moreover, Wang et al. reported that focal hypomethylation is correlated with the frequent overexpression of *HORMAD1* in basal-like breast cancer^93^. They also demonstrated that the epigenetic activation of *HORMAD1* may reduce the sensitivity of basal-like tumors to Rucaparib treatment. Similarly, Tan et al. reported that overexpression of *ART3* in TNBC cells will increase cell proliferation, invasion, and survival in vitro and in vivo, while inverted phenotypes could be observed when *ART3* was knocked down^94^. However, biological functions as well as molecular mechanisms of these genes in breast cancer should be further investigated, as only 8 and 2 papers were available when PubMed was searched with keywords “breast cancer + *HORMAD1*” or “breast cancer + *ART3*,” respectively.

## Conclusion

In conclusion, we highlighted the significance of *CT83* for TNBC and presented a comprehensive bioinformatics strategy for  single-gene analysis in cancer. *CT83* is frequently overexpressed in TNBC and many cancers but silenced in normal non-testis tissues and blood cells. CNV and mutation are not the main genetic alternations of *CT83* in cancer, and *CT83* has no significant correlation with TILs in breast cancer or other cancers. Hypomethylation is probably one of the causes of the abnormal activation of *CT83* in breast cancer. *CT83* is not prognostic in breast cancer but is valuable for the prognosis prediction in KIRP, LIHC, and LUAD. Overexpression of *CT83* in breast cancer may be oncogenic by triggering the activation of signaling associated with the cell cycle.

## Supplementary Information

Supplementary Information.

## Data Availability

The availability of all datasets generated or analyzed during the current study is described in corresponding "Methods" sections, and other data are available from the corresponding author on reasonable request.
