# Supplementary material for: Multiomics analysis reveals CT83 is the most specific gene for triple negative breast cancer and its hypomethylation is oncogenic in breast cancer
Source: Sci Rep. 2021 Jun 9;11:12172. doi: 10.1038/s41598-021-91290-4 (PMC8190062; doi:10.1038/s41598-021-91290-4)
Supplement: Supplementary file 1 — Supplementary Information. [file 41598_2021_91290_MOESM1_ESM.pdf]

Multiomics analysis reveals CT83 is the most specific gene for triple negative breast cancer and its hypomethylation is oncogenic in breast cancer

Chen Chen, Dan Gao, Jinlong Huo, Rui Qu, Youming Guo, Xiaochi Hu, Libo Luo

---

Supplementary Figures and Tables

Figure S1

Expression of CT83 in different types of blood cells

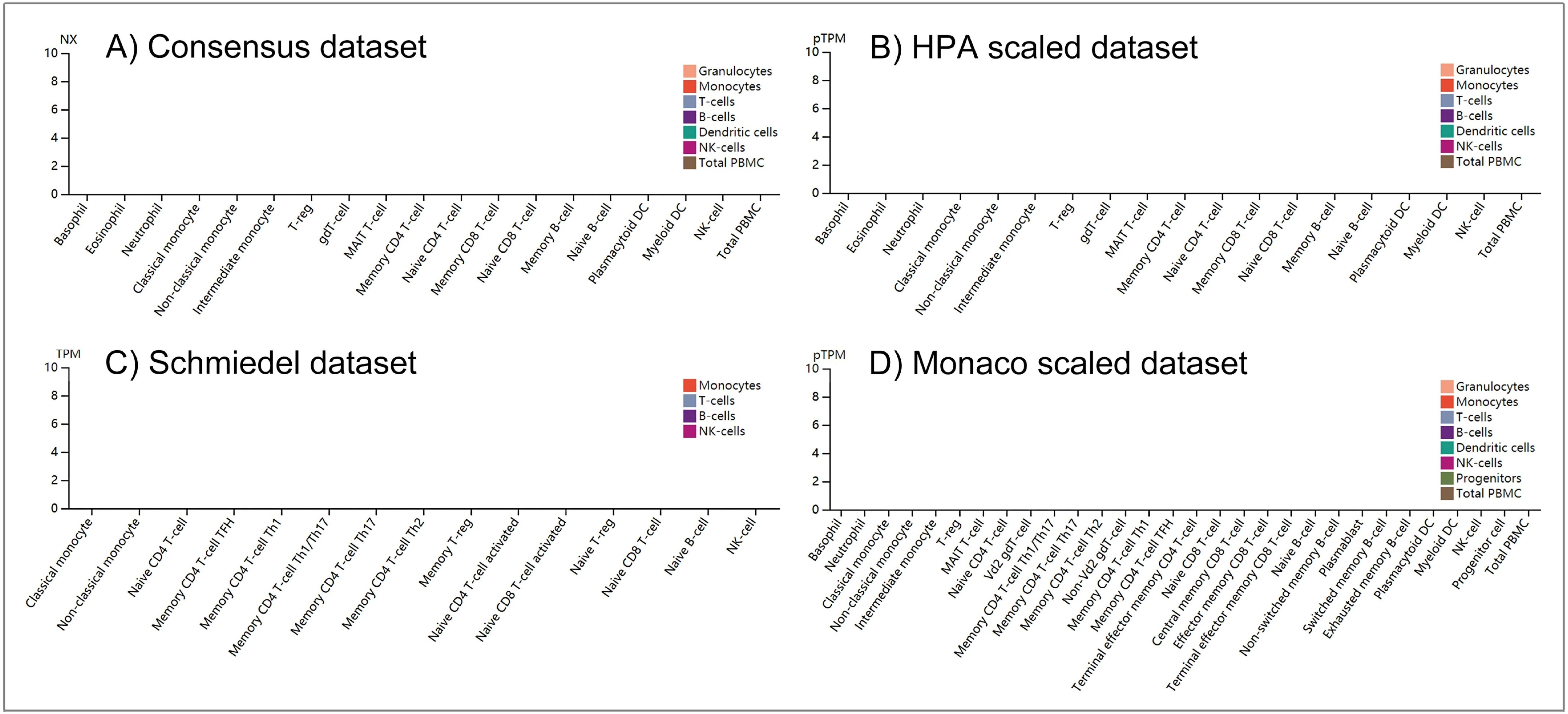

Data were integrated based on the 4 datasets of gene expression in blood cells obtained from the Human Protein Atlas database.

https://www.proteinatlas.org/ENSG00000204019-CT83/blood

Figure S2

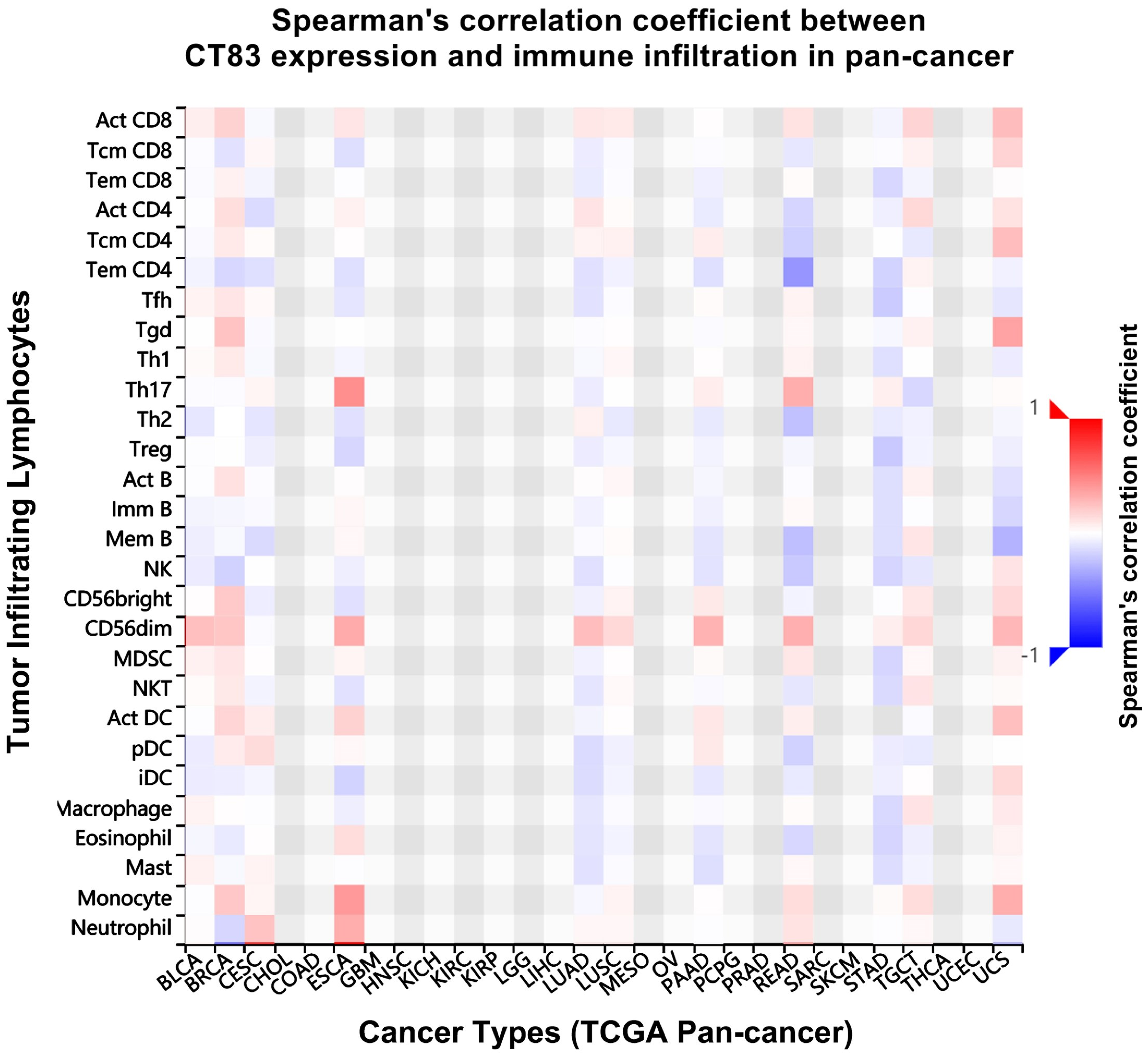
Spearman's correlation coefficient between
CT83 expression and immune infiltration in pan-cancer
Tumor Infiltrating Lymphocytes
Act CD8
Tcm CD8
Tem CD8
Act CD4
Tcm CD4
Tem CD4
Tfh
Tgd
Th1
Th17
Th2
Treg
Act B
Imm B
Mem B
NK
CD56bright
CD56dim
MDSC
NKT
Act DC
pDC
iDC
Macrophage
Eosinophil
Mast
Monocyte
Neutrophil
BLCA
BRCA
CESC
CHOL
COAD
ESCA
GBM
HNSC
KICH
KIRC
KIRP
LGG
LIHC
LUAD
LUSC
MESO
OV
PAAD
PCPG
PRAD
READ
SARC
SKCM
STAD
TGCT
THCA
UCEC
UCS
Cancer Types (TCGA Pan-cancer)
1
-1
Spearman's correlation coefficient

Figure S3

Strategies for screening shared core genes that are usually activated in CT83-positive breast cancer

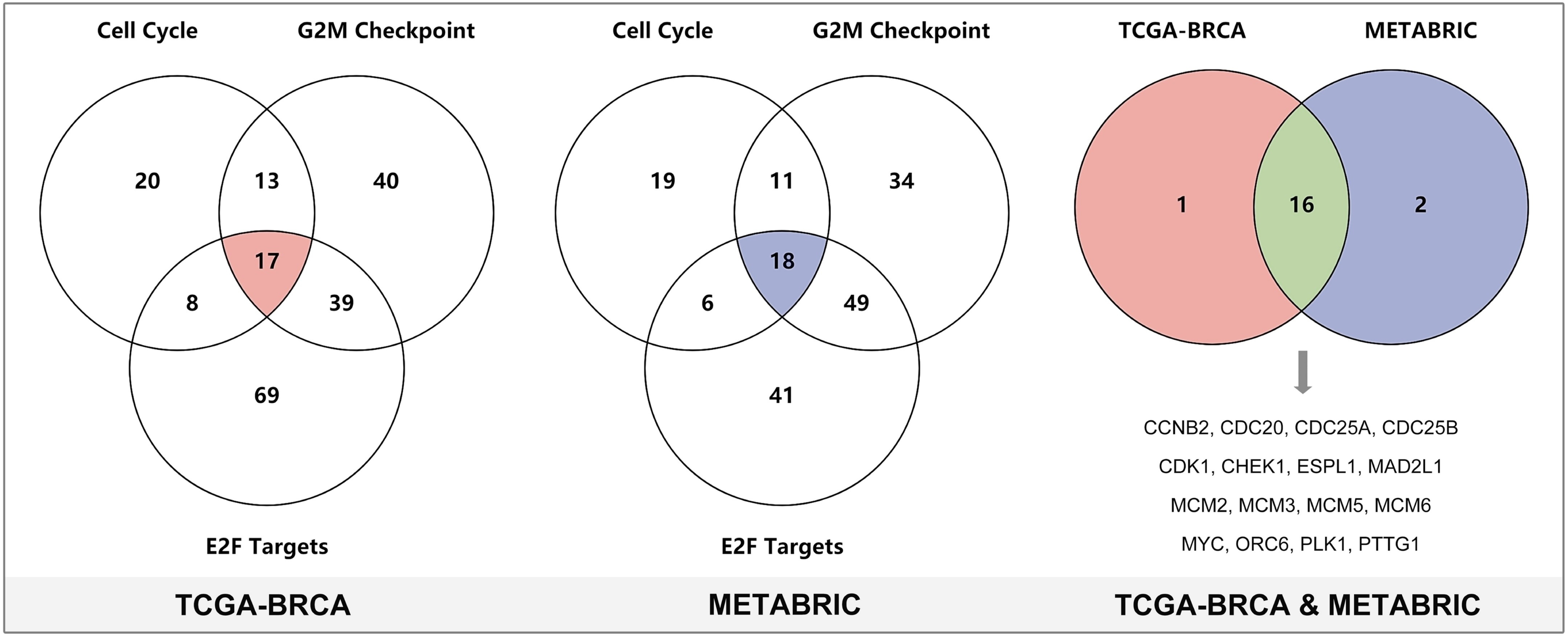

The number in each circle represents the number of **CORE ENRICHED GENES** based on the enrichment scores of GSEA results.

Figure S4

Heatmaps of the Expression of CT83 and its Correlated Genes in Breast Cancer

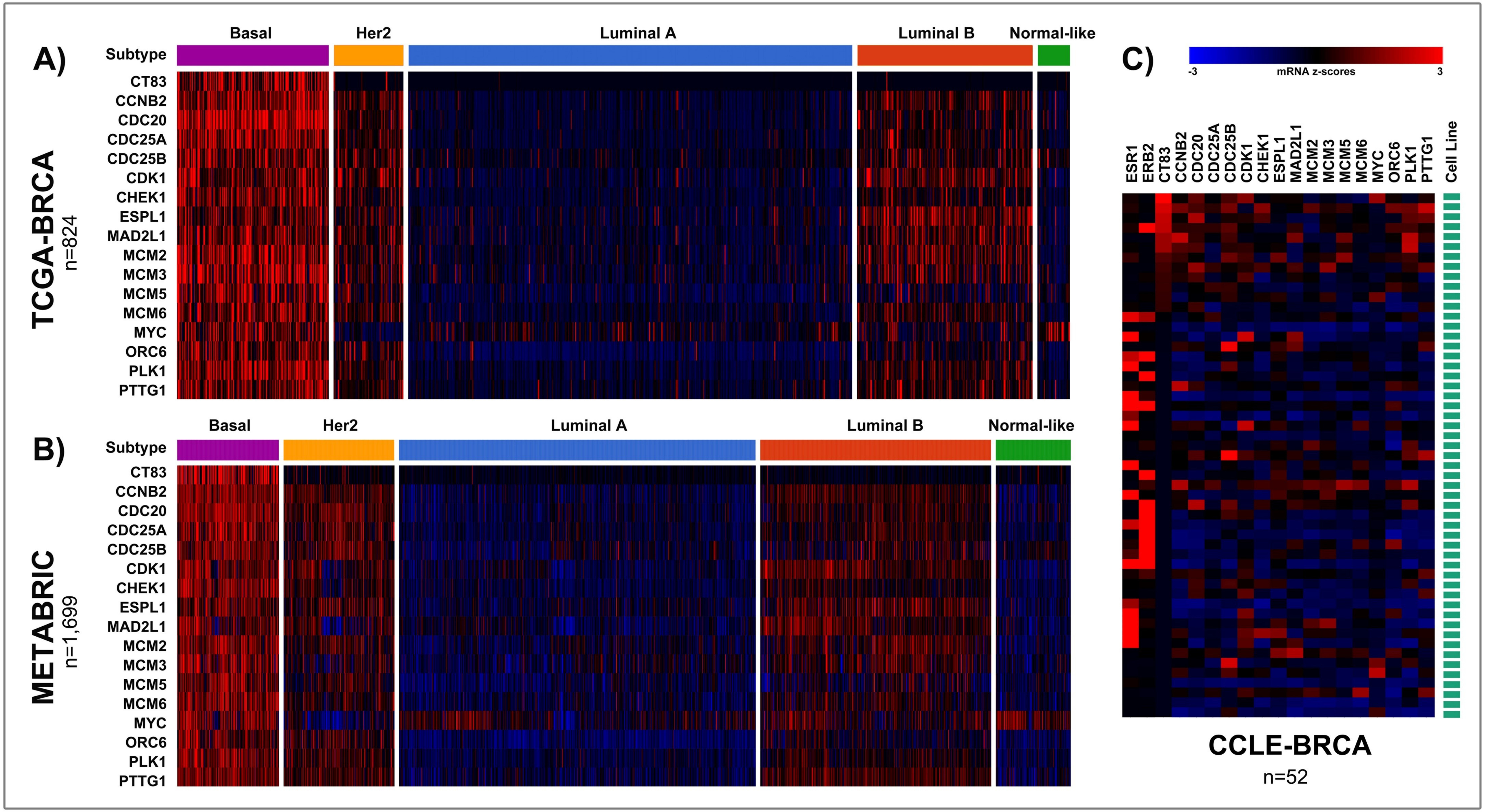

Expression of CT83 and its correlated genes in breast cancer. mRNA expression of CT83 and its 16 correlated genes in breast cancer subtypes according to the RNA-seq data of the A) TCGA-BRCA and B) METABRIC dataset. C) mRNA expression of CT83 and its 16 correlated genes in 71 breast cancer cell lines according to the RNA-seq data of CCLE-BRCA dataset.

Figure S5

Expression of CT83 and Its Correlated Genes in Breast Cancer

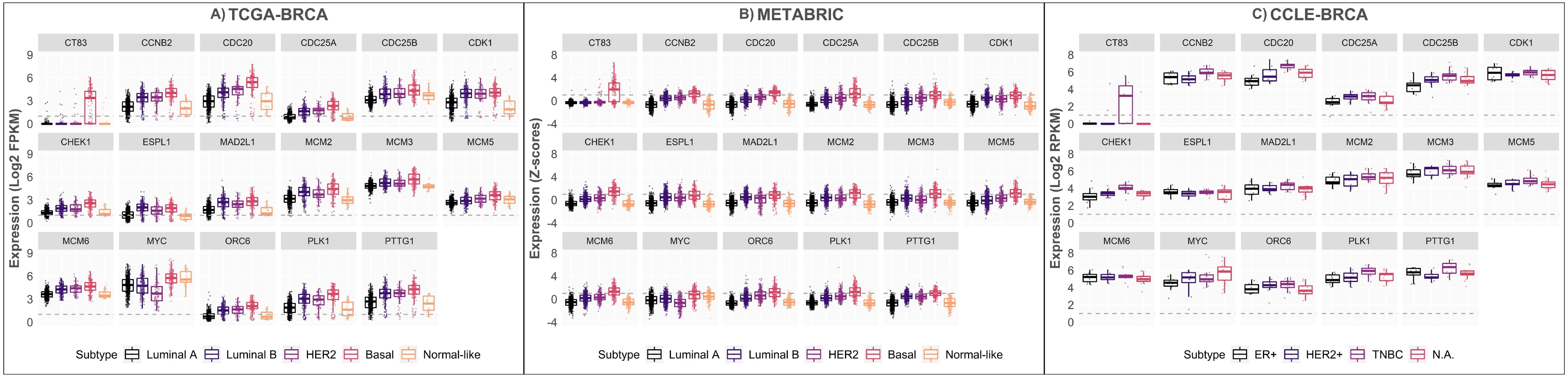

Table S1

Top 10 Significantly Overexpressed Genes in TNBC

	Gene	Luminal A	Luminal B	Her2	Basal	Normal	Max in non-TNBC
TCGA-BRCA (Median FPKM)	CT83	0.00	0.00	0.00	10.44	0.00	0.00
	RP11-19E11.1	0.04	0.02	0.08	6.53	0.18	0.18
	FOXCUT	0.05	0.00	0.04	6.14	0.40	0.40
	HORMAD1	0.03	0.03	0.04	5.77	0.04	0.04
	A2ML1	0.04	0.05	0.40	4.82	0.12	0.40
	GAL	0.31	0.33	0.70	4.05	0.37	0.70
	PCSK1N	0.25	0.11	0.31	3.81	0.23	0.31
	ARL9	0.39	0.53	0.66	3.71	0.67	0.67
	GJB3	0.19	0.12	0.20	3.62	0.49	0.49
	ART3	0.03	0.02	0.05	3.09	0.12	0.12
METABRIC (Median Z-scores)	VGLL1	-0.36	-0.42	-0.14	2.46	-0.09	-0.09
	CT83	-0.26	-0.25	-0.18	2.28	-0.25	-0.18
	UGT8	-0.32	-0.32	-0.28	2.26	-0.01	-0.01
	CDKN2A	-0.30	-0.20	-0.15	2.20	-0.39	-0.15
	ATL2	-0.41	-0.15	-0.09	2.06	-0.07	-0.07
	ACTR3B	-0.30	-0.34	-0.44	1.92	-0.12	-0.12
	HORMAD1	-0.29	-0.27	-0.22	1.91	-0.25	-0.22
	DSC2	-0.41	-0.33	-0.05	1.83	0.00	0.00
	ART3	-0.29	-0.30	-0.24	1.77	-0.23	-0.23
	KRT16P3	-0.35	-0.43	-0.11	1.75	-0.15	-0.11

Table S2

mRNA Expression, DNA Copy Number, and Copy Number Variation of CT83 in 59 Types of Breast (Cancer) Cell Lines

Cell Line	ATCC #	Pathology	CT83	CT83	CT83	BrC. Subtype	BrC. Subtype	ESR1	ER	PgR	PgR	ERBB2	ERBB2
			CCLE mRNA, RPKM	CCLE Copy Number	CCLE CNV	Smith et al. Subtype	Smith et al. PAM50	CCLE mRNA, RPKM	Smith et al. Protein	CCLE mRNA, RPKM	Smith et al. Protein	CCLE mRNA, RPKM	Smith et al. Protein
AU565	CRL-2351	Metastasis	0	-0.4949	NC	HER2+	HER2	0	-	0	-	1519	+++
BT-20	HTB-19	Primary	0	1.0873	AMP	TNBC	Basal A	1	-	0	-	33	+
BT-474	HTB-20	Primary	0	-0.5108	NC	HER2+	HER2	7	+	44	+++	796	+++
BT-483	HTB-121	Primary	0	0.0075	NC	ER+	Luminal B	12	+	0	-	133	++
BT-549	HTB-122	Primary	0	0.6944	NC	TNBC	Basal B	0	-	0	-	8	+/-
CAL-120		Metastasis	0	0.0098	NC			0		0		11	
CAL-148		Metastasis	0	0.0016	NC			0		0		111	
CAL-51		Metastasis	0	-0.0320	NC			0		0		36	
CAL-85-1		Primary	0	0.7327	AMP			0		0		24	
CAMA-1	HTB-21	Metastasis	0	-0.0062	NC	ER+	Luminal A	9	+	6	+	36	+
DU4475	HTB-123	Metastasis	0	0.0056	NC	TNBC		0	-	0	-	4	-
EFM-19		Metastasis	0	0.1858	NC			18		14		87	
EFM-192A		Metastasis	0	0.0012	NC			2		0		1518	
HCC1143	HCC1143	Primary	12	0.8862	AMP			2		0		16	
HCC1187	CRL-2322	Primary	0	0.7196	AMP	TNBC	Basal A	0	-	0	-	49	+
HCC1395	CRL-2324	Primary	20	0.5679	NC	TNBC	Basal B	0	-	0	-	7	+/-
HCC1419	CRL-2326	Primary	0	0.0227	NC	HER2+	HER2	5	+	0	-	1529	+++
HCC1428	CRL-2327	Metastasis	0	-0.0451	NC	ER+	Luminal A	24	++	11	-	19	+/-
HCC1500	CRL-2329	Primary	0	0.0527	NC	ER+	Luminal A	38	+++	8	+	20	+/-
HCC1569	CRL-2330	Primary	0	-0.0851	NC	HER2+	HER2	0	-	0	-	998	+++
HCC1599	CRL-2331	Primary	46	1.1759	AMP	TNBC	Basal A	1	-	0	-	22	-
HCC1806	CRL-2335	Primary	8	-0.0001	NC	TNBC	Basal A	1	-	0	-	20	-
HCC1937	CRL-2336	Primary	26	-0.3094	NC	TNBC	Basal A	1	-	0	-	23	-
HCC1954	CRL-2338	Primary	24	-0.0132	NC	HER2+	HER2	1	-	0	-	1714	+++
HCC202	CRL-2316	Primary	0	-1.1648	DEL	HER2+	HER2	0	-	0	-	1073	+++
HCC2157	CRL-2340	Primary	8	-0.2729	NC	TNBC	Basal A	0	-	0	-	47	+
HCC2218	CRL-2343	Primary	0	0.0093	NC	HER2+	HER2	0	-	0	-	1157	+++
HCC38	CRL-2314	Primary	9	1.0248	AMP	TNBC	Basal B	0	-	0	-	58	+
HCC70	CRL-2315	Primary	19	0.2916	NC	TNBC	Basal A	1	-	0	-	19	+/-
HDQ-P1		Primary	0	0.0366	NC			1		0		27	
HMC-1-8		Metastasis	0	0.0934	NC			0		0		6	
HMEL			0	0.0037	NC			0		0		20	
Hs 274.T	CRL-7222	Primary	0	0.0066	NC			0		0		14	
JIMT-1		Metastasis	0	0.1292	NC			1		0		179	
KPL-1		Metastasis	0	0.3295	NC			9		0		26	
MCF7	HTB-22	Metastasis	0	0.4003	NC	ER+	Luminal A	18	++	4	+/-	24	-
MDA-MB-134-VI	HTB-23	Metastasis	0	0.0308	NC	ER+	Luminal A	39	++	1	-	11	-
MDA-MB-157	HTB-24	Metastasis	0	-0.1164	NC	TNBC	Basal B	0	+/-	0	-	10	-
MDA-MB-175-VII	HTB-25	Metastasis	0	0.0006	NC	ER+	Luminal B	4	+/-	0	-	94	+
MDA-MB-231	HTB-26	Metastasis	10	-0.2376	NC	TNBC	Basal B	0	-	0	-	12	-
MDA-MB-361	HTB-27	Metastasis	2	-0.7120	DEL	HER2+	HER2	6	++	1	-	362	++
MDA-MB-415	HTB-128	Metastasis	0	0.0230	NC	ER+	Luminal A	4	+/-	1	-	31	+/-
MDA-MB-436	HTB-130	Metastasis	9	0.1899	NC	TNBC	Basal B	0	-	0	-	7	-
MDA-MB-453	HTB-131	Metastasis	0	-0.1366	NC	TNBC	Basal A	0	-	0	-	148	+
MDA-MB-468	HTB-132	Metastasis	24	0.2932	NC	TNBC	Basal A	1	-	0	-	11	-
SK-BR-3	HTB-30	Metastasis	0	-0.5219	NC	HER2+	HER2	0	-	0	-	1011	+++
T-47D	HTB-133	Metastasis	0	-0.3782	NC	ER+	Luminal A	19	++	70	+++	43	+/-
UACC-812	CRL-1897	Primary	0	0.1306	NC	HER2+	HER2	9	-	11	-	1162	+++
UACC-893	CRL-1902	Primary	0	0.3740	NC	HER2+	HER2	1	-	0	-	1652	+++
ZR-75-1	CRL-1500	Metastasis	0	0.0227	NC	ER+	Luminal A	12	+/-	20	+/-	50	+/-
ZR-75-30	CRL-1504	Metastasis	0	0.0143	NC	HER2+	HER2	5	-	0	-	1401	+++
184B5	CRL-8799					N			-		-		-
MCF-10A	CRL-10317					N	Basal B		-		-		-
MCF-10F	CRL-10318					N	Basal B		-		-		-
MCF-12A	CRL-10782					N			-		-		-
Hs 578T	HTB-126	Primary	0	-0.1530	NC	TNBC	Basal B		-		-		-
MDA-kb2	CRL-2713					TNBC	Basal A		-		-		++

Table S3

CT83 Mutations in Cancer

Study	Sample ID	Cancer Type	Protein Change	Mutation Type	Variant Type	Copy #	Chromosome	Start Pos	End Pos	Ref	Var
Skin Cutaneous Melanoma (TCGA, Firehose Legacy)	TCGA-EE-A2MJ-06	Cutaneous Melanoma	E30K	Missense_Mutation	SNP	diploid	23	115593162	115593162	C	T
Skin Cutaneous Melanoma (TCGA, Firehose Legacy)	TCGA-EE-A3J5-06	Cutaneous Melanoma	R79Q	Missense_Mutation	SNP	diploid	23	115593014	115593014	C	T
Stomach Adenocarcinoma (TCGA, Firehose Legacy)	TCGA-BR-6452-01	Stomach Adenocarcinoma	S107P	Missense_Mutation	SNP	diploid	23	115592931	115592931	A	G
Lung Adenocarcinoma (TCGA, Firehose Legacy)	TCGA-44-2656-01	Lung Adenocarcinoma	S46Y	Missense_Mutation	SNP	diploid	23	115593113	115593113	G	T
Lung Squamous Cell Carcinoma (TCGA, Firehose Legacy)	TCGA-18-3414-01	Lung Squamous Cell Carcinoma	D61Y	Missense_Mutation	SNP	diploid	23	115593069	115593069	C	A
Lung Squamous Cell Carcinoma (TCGA, Firehose Legacy)	TCGA-43-3394-01	Lung Squamous Cell Carcinoma	R23C	Missense_Mutation	SNP	diploid	23	115593951	115593951	G	A
Skin Cutaneous Melanoma (TCGA, Firehose Legacy)	TCGA-EE-A29L-06	Cutaneous Melanoma	S35L	Missense_Mutation	SNP	diploid	23	115593146	115593146	G	A
Breast Invasive Carcinoma (TCGA, Firehose Legacy)	TCGA-A2-A04T-01	Breast Invasive Ductal Carcinoma	N34H	Missense_Mutation	SNP	gain	23	115593150	115593150	T	G
Thyroid Carcinoma (TCGA, Firehose Legacy)	TCGA-EM-A2OV-01	Follicular Thyroid Cancer	G102S	Missense_Mutation	SNP	diploid	23	115592946	115592946	C	T
Prostate Adenocarcinoma (TCGA, Firehose Legacy)	TCGA-XK-AAIW-01	Prostate Adenocarcinoma	R22H	Missense_Mutation	SNP	diploid	23	115593953	115593953	C	T
Colorectal Adenocarcinoma (TCGA, Firehose Legacy)	TCGA-AA-3864-01	Colon Adenocarcinoma	E88G	Missense_Mutation	SNP	diploid	23	115592987	115592987	T	C
Skin Cutaneous Melanoma (TCGA, Firehose Legacy)	TCGA-ER-A19G-06	Cutaneous Melanoma	R22C	Missense_Mutation	SNP	gain	23	115593954	115593954	G	A
Skin Cutaneous Melanoma (TCGA, Firehose Legacy)	TCGA-EE-A20H-06	Cutaneous Melanoma	E95K	Missense_Mutation	SNP	diploid	23	115592967	115592967	C	T
Adrenocortical Carcinoma (TCGA, Firehose Legacy)	TCGA-OR-A5KB-01	Adrenocortical Carcinoma	A8T	Missense_Mutation	SNP	gain	23	115593996	115593996	C	T
Cervical Squamous Cell Carcinoma and Endocervical Adenocarcinoma (TCGA, Firehose Legacy)	TCGA-FU-A3HZ-01	Cervical Squamous Cell Carcinoma	N83T	Missense_Mutation	SNP	diploid	23	115593002	115593002	T	G
Cancer Cell Line Encyclopedia (Broad, 2019)	HEC1_ENDOMETRIUM	Mixed Cancer Types	R22H	Missense_Mutation	SNP		23	115593953	115593953	C	T
Cancer Cell Line Encyclopedia (Broad, 2019)	SF268_CENTRAL_NERVOUS_SYSTEM	Mixed Cancer Types	N68D	Missense_Mutation	SNP	diploid	23	115593048	115593048	T	C
Cancer Cell Line Encyclopedia (Broad, 2019)	HEC251_ENDOMETRIUM	Mixed Cancer Types	D61N	Missense_Mutation	SNP	diploid	23	115593069	115593069	C	T
Cancer Cell Line Encyclopedia (Broad, 2019)	KARPAS1106P_HAEMATOPOIETIC_AND_LYMPHOID_TISSUE	Mixed Cancer Types	D61N	Missense_Mutation	SNP		23	115593069	115593069	C	T
Cancer Cell Line Encyclopedia (Broad, 2019)	K2_SKIN	Mixed Cancer Types	D61N	Missense_Mutation	SNP		23	115593069	115593069	C	T
Cancer Cell Line Encyclopedia (Broad, 2019)	NCIH1944_LUNG	Mixed Cancer Types	N89K	Missense_Mutation	SNP	diploid	23	115592983	115592983	G	T
Cancer Cell Line Encyclopedia (Broad, 2019)	GCT_SOFT_TISSUE	Mixed Cancer Types	D65N	Missense_Mutation	SNP	diploid	23	115593057	115593057	C	T
Cancer Cell Line Encyclopedia (Broad, 2019)	RXF393_KIDNEY	Mixed Cancer Types	L48*	Nonsense_Mutation	SNP		23	115593107	115593107	A	C

Table S4

CT83 and Immune Infiltration in Breast Cancer

Infiltrates	Breast Cancer n = 1,100			Luminal A n = 568		Luminal B n = 219		Her2 n = 82		Basal n = 191	
	Spearman's	P		Spearman's	P	Spearman's	P	Spearman's	P	Spearman's	P
B cell memory_CIBERSORT	0.0676	0.3252		0.0286	0.7905	0.0586	0.7464	-0.1127	0.6910	-0.1817	0.2385
B cell memory_CIBERSORT-ABS	0.0747	0.2412		0.0329	0.7560	0.0531	0.7660	-0.1213	0.6601	-0.1612	0.3252
B cell memory_XCELL	0.2129	0.0000	*	0.1068	0.2363	-0.0767	0.6521	-0.0103	0.9935	0.0227	0.9226
B cell naive_CIBERSORT	-0.0341	0.6410		0.0442	0.6601	-0.0900	0.6035	0.1920	0.5000	0.1570	0.3373
B cell naive_CIBERSORT-ABS	0.0128	0.8933		0.0865	0.3510	-0.0754	0.6569	0.1854	0.5116	0.1178	0.5116
B cell naive_XCELL	0.1982	0.0000	*	0.1061	0.2367	0.0639	0.7211	-0.0063	0.9953	0.1259	0.4884
B cell plasma_CIBERSORT	0.0614	0.3574		0.0603	0.5620	0.0408	0.8238	0.0877	0.7660	0.1438	0.3700
B cell plasma_CIBERSORT-ABS	0.0829	0.1756		0.0540	0.6044	0.0746	0.6599	0.1637	0.5620	0.0954	0.6018
B cell plasma_XCELL	0.2131	0.0000	*	0.1190	0.1482	0.1349	0.3904	0.0972	0.7464	0.0428	0.8238
B cell_EPIC	0.1479	0.0003	*	0.1008	0.2572	0.1036	0.5343	0.0263	0.9397	-0.0304	0.8933
B cell_MCPCOUNTER	0.0819	0.1814		0.0910	0.3373	0.0753	0.6569	0.0614	0.8538	0.0012	0.9954
B cell_QUANTISEQ	0.0700	0.2826		0.0648	0.5280	0.0074	0.9919	-0.0821	0.7808	0.0515	0.7854
B cell_TIMER	-0.0711	0.2739		0.0289	0.7887	-0.0280	0.8933	0.0005	0.9982	0.0236	0.9211
B cell_XCELL	0.2068	0.0000	*	0.1258	0.1083	0.0219	0.9226	0.0517	0.8830	-0.0172	0.9397
Cancer associated fibroblast_EPIC	-0.2576	0.0000	*	-0.0911	0.2810	0.0440	0.7782	-0.2374	0.3032	-0.0389	0.7980
Cancer associated fibroblast_MCPCOUNTER	-0.2196	0.0000	*	-0.0843	0.3236	0.0537	0.7284	-0.2441	0.2810	-0.0683	0.6853
Cancer associated fibroblast_TIDE	-0.2187	0.0000	*	-0.1095	0.1767	0.0692	0.6475	-0.2114	0.3633	-0.0094	0.9492
Cancer associated fibroblast_XCELL	-0.2427	0.0000	*	-0.0060	0.9459	0.0435	0.7782	-0.2104	0.3633	-0.1757	0.2123
Class-switched memory B cell_XCELL	0.0509	0.5000		0.0976	0.2801	0.0484	0.7867	0.0828	0.7806	-0.1198	0.5116
Common lymphoid progenitor_XCELL	-0.1292	0.0016	*	-0.0564	0.5122	-0.0532	0.7624	-0.0276	0.9103	-0.0007	0.9928
Common myeloid progenitor_XCELL	-0.2110	0.0000	*	-0.0629	0.4537	-0.0274	0.8708	-0.0551	0.8686	0.0475	0.8065
Endothelial cell_EPIC	-0.2213	0.0000	*	-0.0873	0.2968	-0.0536	0.8119	-0.0961	0.8119	-0.0263	0.9371
Endothelial cell_MCPCOUNTER	-0.1691	0.0000	*	-0.0971	0.2179	-0.0537	0.8119	-0.1368	0.6903	-0.0515	0.8232
Endothelial cell_XCELL	-0.2009	0.0000	*	-0.0830	0.3175	-0.0563	0.8119	-0.0903	0.8119	-0.0462	0.8527
Eosinophil_CIBERSORT	-0.0275	0.8119		-0.0192	0.9162	-0.0422	0.8527			-0.1369	0.3198
Eosinophil_CIBERSORT-ABS	-0.0275	0.8119		-0.0192	0.9162	-0.0422	0.8527			-0.1378	0.3175
Eosinophil_XCELL	0.0878	0.0790		0.0582	0.5895	0.0909	0.6110	-0.0455	0.9303	0.0944	0.6110
Granulocyte-monocyte progenitor_XCELL	-0.0317	0.5957		0.0413	0.6201	0.0293	0.8908	-0.1000	0.6632	-0.0110	0.9229
Hematopoietic stem cell_XCELL	-0.2315	0.0000	*	-0.0444	0.5957	-0.0792	0.5957	-0.1869	0.3899	-0.0186	0.9092
Macrophage M0_CIBERSORT	0.1640	0.0000	*	0.0358	0.7058	-0.0575	0.7147	-0.0020	0.9986	-0.0712	0.6387
Macrophage M0_CIBERSORT-ABS	0.2170	0.0000	*	0.0537	0.5305	-0.0124	0.9429	0.0888	0.7423	-0.0853	0.5694
Macrophage M1_CIBERSORT	0.1959	0.0000	*	0.0240	0.8285	0.0500	0.7588	0.1274	0.5858	0.1770	0.2002
Macrophage M1_CIBERSORT-ABS	0.1990	0.0000	*	0.0396	0.6572	0.0744	0.5918	0.0427	0.8922	0.0854	0.5694
Macrophage M1_QUANTISEQ	0.0686	0.2240		-0.0364	0.7034	-0.0220	0.9148	0.0139	0.9616	0.0062	0.9755
Macrophage M1_XCELL	0.2063	0.0000	*	0.0276	0.7872	0.0911	0.5117	0.0617	0.8389	-0.0240	0.9136
Macrophage M2_CIBERSORT	-0.2600	0.0000	*	-0.0995	0.2107	-0.0612	0.6884	-0.1198	0.6033	0.0306	0.8748
Macrophage M2_CIBERSORT-ABS	-0.0706	0.2126		-0.0499	0.5694	0.0297	0.8697	-0.0492	0.8697	-0.0514	0.7687
Macrophage M2_QUANTISEQ	-0.3623	0.0000	*	-0.0594	0.4821	-0.0237	0.9052	-0.0260	0.9304	-0.1065	0.4580
Macrophage M2_TIDE	-0.2322	0.0000	*	-0.0183	0.8697	-0.0758	0.5918	-0.0088	0.9768	-0.0825	0.5799
Macrophage M2_XCELL	-0.1594	0.0000	*	-0.0573	0.4955	0.0594	0.7057	-0.0154	0.9601	-0.1157	0.4061
Macrophage/Monocyte_MCPCOUNTER	0.3279	0.0000	*	-0.0902	1.2406	0.2366	0.7745	0.0608	1.8735	-0.2386	0.8024
Macrophage_EPIC	0.0582	0.3206		-0.0402	0.6496	-0.0104	0.9556	0.0074	0.9818	-0.0529	0.7588
Macrophage_TIMER	-0.2167	0.0000	*	-0.1276	0.0750	-0.0345	0.8498	0.0145	0.9616	-0.0939	0.5234
Macrophage_XCELL	0.0482	0.4061		-0.0257	0.8098	0.0359	0.8433	-0.0120	0.9694	-0.0921	0.5360
Mast cell activated_CIBERSORT	-0.3533	0.0000	*	-0.1116	0.1606	0.0522	0.7351	-0.0825	0.7462	-0.1214	0.4048
Mast cell activated_CIBERSORT-ABS	-0.3057	0.0000	*	-0.0895	0.2979	0.0822	0.5625	-0.1002	0.6949	-0.1216	0.4048
Mast cell resting_CIBERSORT	0.1194	0.0035	*	-0.0213	0.7980	-0.0211	0.8756	0.0019	0.9877	0.0155	0.9104
Mast cell resting_CIBERSORT-ABS	0.1283	0.0013	*	-0.0202	0.8126	-0.0234	0.8670	0.0102	0.9551	0.0127	0.9323
Mast cell_XCELL	-0.2861	0.0000	*	-0.0627	0.4414	0.0319	0.8233	-0.0996	0.6952	-0.1052	0.4488
MDSC_TIDE	0.2950	0.0000	*	0.0003	0.9947	-0.0068	0.9474	0.0358	0.8872	0.1367	0.3336
Monocyte_CIBERSORT	-0.0635	0.3470		0.0194	0.8936	-0.0609	0.7248	-0.1464	0.5567	0.0426	0.8510
Monocyte_CIBERSORT-ABS	-0.0274	0.7121		0.0290	0.7887	-0.0505	0.7771	-0.1893	0.3995	0.0267	0.9311
Monocyte_MCPCOUNTER	0.1639	0.0000	*	-0.0451	0.6488	0.1183	0.3854	0.0304	0.9450	-0.1193	0.3995
Monocyte_QUANTISEQ	0.1328	0.0013	*	0.0074	0.9615	-0.0066	0.9753	0.0393	0.9340	-0.0873	0.5882
Monocyte_XCELL	0.1290	0.0020	*	0.0864	0.3486	-0.0224	0.9378	-0.1165	0.6708	-0.0978	0.5489
Myeloid dendritic cell activated_CIBERSORT	0.1827	0.0000	*	0.0317	0.7795	0.1201	0.5048	0.0953	0.7777	0.2090	0.0891
Myeloid dendritic cell activated_CIBERSORT-ABS	0.1872	0.0000	*	0.0314	0.7795	0.1273	0.4734	0.1007	0.7777	0.2027	0.1080
Myeloid dendritic cell activated_XCELL	0.1663	0.0000	*	0.0478	0.6820	0.0590	0.7777	0.1176	0.7320	0.0163	0.9351
Myeloid dendritic cell resting_CIBERSORT	-0.0470	0.5562		-0.0222	0.8681	-0.0315	0.8811	0.2073	0.4775	-0.1359	0.4577
Myeloid dendritic cell resting_CIBERSORT-ABS	-0.0233	0.7795		-0.0075	0.9560	-0.0196	0.9162	0.2197	0.4269	-0.1173	0.5468
Myeloid dendritic cell_MCPCOUNTER	0.0133	0.8811		0.0003	0.9968	0.0516	0.7795	0.0964	0.7777	-0.1816	0.1885
Myeloid dendritic cell_QUANTISEQ	0.0613	0.3911		-0.0213	0.8732	0.0130	0.9505	0.0168	0.9685	0.0025	0.9935
Myeloid dendritic cell_TIMER	0.1792	0.0000	*	0.0047	0.9754	0.0631	0.7777	-0.0031	0.9942	-0.0356	0.8776
Myeloid dendritic cell_XCELL	0.1884	0.0000	*	0.0378	0.7777	0.1441	0.3648	0.1833	0.5468	-0.1433	0.4110
Neutrophil_CIBERSORT	0.0316	0.6651		-0.0678	0.4199	0.0923	0.5489	0.1596	0.5219	-0.0257	0.9330
Neutrophil_CIBERSORT-ABS	0.0399	0.5489		-0.0697	0.3995	0.1020	0.5023	0.1624	0.5214	-0.0214	0.9390
Neutrophil_MCPCOUNTER	-0.2819	0.0000	*	-0.0760	0.3694	-0.0391	0.8677	-0.1153	0.6770	-0.0805	0.6465
Neutrophil_QUANTISEQ	0.2257	0.0000	*	-0.1174	0.1187	0.0252	0.9311	0.2644	0.2600	0.0676	0.7010
Neutrophil_TIMER	0.2695	0.0000	*	-0.0519	0.5699	0.0137	0.9596	0.0365	0.9378	-0.0060	0.9793
Neutrophil_XCELL	0.0218	0.7771		-0.0300	0.7771	-0.0913	0.5489	0.1942	0.3854	-0.1765	0.2303
NK cell activated_CIBERSORT	0.1041	0.0257	*	0.0349	0.7777	0.0675	0.7445	0.0850	0.7795	0.1080	0.5562
NK cell activated_CIBERSORT-ABS	0.1327	0.0013	*	0.0339	0.7784	0.0855	0.6434	0.0317	0.9162	0.0933	0.6346
NK cell resting_CIBERSORT	0.0004	0.9953		-0.0368	0.7777	-0.0590	0.7777	0.0077	0.9873	-0.0582	0.7784
NK cell resting_CIBERSORT-ABS	0.0048	0.9631		-0.0352	0.7777	-0.0617	0.7777	0.0152	0.9733	-0.0541	0.7795
NK cell_EPIC	0.1074	0.0184	*	0.0185	0.8811	0.0686	0.7401	0.0931	0.7784	0.0427	0.8480
NK cell_MCPCOUNTER	0.1020	0.0316	*	0.0053	0.9735	0.0537	0.7795	0.1353	0.6575	0.0279	0.8960
NK cell_QUANTISEQ	0.1152	0.0087	*	-0.0017	0.9929	0.0343	0.8751	-0.0388	0.9044	-0.0873	0.6575
NK cell_XCELL	0.0260	0.7777		0.0122	0.9162	0.1080	0.5562	-0.0054	0.9925	0.0277	0.8960
Plasmacytoid dendritic cell_XCELL	0.2178	0.0000	*	0.0317	0.7795	0.0662	0.7525	0.0523	0.8811	0.0535	0.7795
T cell CD4+ (non-regulatory)_QUANTISEQ	-0.3387	0.0000	*	0.0282	0.8742	-0.1699	0.2012	0.0075	0.9874	-0.1268	0.4663
T cell CD4+ (non-regulatory)_XCELL	0.0322	0.7552		0.0297	0.8645	0.0326	0.9033	0.0995	0.8396	-0.0629	0.8396
T cell CD4+ central memory_XCELL	-0.0896	0.0831		0.0671	0.4909	-0.1847	0.1396	-0.0504	0.9033	-0.0645	0.8396
T cell CD4+ effector memory_XCELL	-0.1514	0.0001	*	0.0696	0.4866	-0.0738	0.7551	-0.2682	0.2274	-0.0617	0.8396
T cell CD4+ memory activated_CIBERSORT	0.2119	0.0000	*	0.1259	0.0830	0.0604	0.8396	0.0684	0.8837	-0.1259	0.4749
T cell CD4+ memory activated_CIBERSORT-ABS	0.2126	0.0000	*	0.1268	0.0807	0.0632	0.8334	0.0808	0.8645	-0.1354	0.4530
T cell CD4+ memory resting_CIBERSORT	-0.0285	0.8245		0.0852	0.3652	0.0291	0.9036	-0.0890	0.8487	0.0230	0.9258
T cell CD4+ memory resting_CIBERSORT-ABS	0.0365	0.6574		0.0347	0.8397	0.0543	0.8487	-0.0264	0.9341	-0.0052	0.9874
T cell CD4+ memory_XCELL	0.1392	0.0006	*	0.0434	0.7618	0.0106	0.9559	0.0472	0.9036	-0.0601	0.8397
T cell CD4+ naive_CIBERSORT-ABS	-0.0242	0.8478		-0.0180	0.9033	-0.0238	0.9177			-0.0360	0.9033
T cell CD4+ naive_XCELL	0.0656	0.3145		0.0652	0.5142	-0.0370	0.9033	0.1273	0.7186	-0.1318	0.4541
T cell CD4+ Th1_XCELL	0.1949	0.0000	*	0.0437	0.7618	-0.0381	0.9033	0.1821	0.4909	0.0196	0.9316
T cell CD4+ Th2_XCELL	0.4213	0.0000	*	-0.0082	0.9399	0.2053	0.0830	0.2031	0.4570	0.0380	0.9033
T cell CD4+_EPIC	-0.1390	0.0006	*	-0.0147	0.9177	-0.0259	0.9121	-0.0906	0.8487	-0.1453	0.3678
T cell CD4+_TIMER	0.1881	0.0000	*	0.1198	0.0979	-0.0188	0.9316	-0.0020	0.9960	0.1186	0.4878
T cell CD8+ central memory_XCELL	0.1234	0.0059	*	0.0854	0.3065	0.0571	0.7788	0.1106	0.7063	-0.0306	0.9031
T cell CD8+ effector memory_XCELL	0.0890	0.1171		0.0303	0.7981	-0.0040	0.9900	-0.0615	0.8683	0.0452	0.8303
T cell CD8+ naive_XCELL	0.0326	0.6635		0.0979	0.2406	-0.0201	0.9305	0.0840	0.7971	-0.0526	0.7981
T cell CD8+_CIBERSORT	0.0301	0.6977		0.0676	0.4403	0.0069	0.9758	0.1859	0.4389	0.0011	0.9962
T cell CD8+_CIBERSORT-ABS	0.0888	0.1171		0.0535	0.5795	0.0354	0.8806	0.1242	0.6613	-0.0007	0.9962
T cell CD8+_EPIC	0.1108	0.0215	*	0.0541	0.5795	0.0266	0.9223	0.1309	0.6239	0.0335	0.8921
T cell CD8+_MCPCOUNTER	0.2154	0.0000	*	0.0792	0.3859	0.0460	0.8173	0.1681	0.4694	0.0746	0.6779
T cell CD8+_QUANTISEQ	0.1589	0.0001	*	0.0888	0.2875	0.0454	0.8180	0.1647	0.4853	0.0665	0.7355
T cell CD8+_TIMER	-0.2054	0.0000	*	-0.0600	0.4968	-0.0175	0.9540	0.0408	0.9223	-0.1224	0.4285
T cell CD8+_XCELL	0.1319	0.0023	*	0.0944	0.2511	-0.0880	0.5795	-0.0307	0.9436	-0.0087	0.9733
T cell follicular helper_CIBERSORT	0.1754	0.0000	*	0.0045	0.9438	-0.0508	0.7326	0.1079	0.6549	0.0747	0.6359
T cell follicular helper_CIBERSORT-ABS	0.2156	0.0000	*	0.0125	0.8872	0.0230	0.8822	0.1074	0.6549	0.0500	0.7495
T cell gamma delta_CIBERSORT	-0.0182	0.7711		0.0473	0.5827	0.0895	0.5124	0.1256	0.5932	-0.0649	0.6641
T cell gamma delta_CIBERSORT-ABS	-0.0058	0.9349		0.0543	0.5124	0.1032	0.4346	0.1264	0.5914	-0.0650	0.6641
T cell gamma delta_XCELL	-0.0589	0.3038		-0.0451	0.6135	-0.0262	0.8729	0.1686	0.4346	0.0097	0.9414
T cell NK_XCELL	-0.0107	0.8817		0.0934	0.2261	-0.1157	0.3760	0.0052	0.9802	0.1477	0.2681
T cell regulatory (Tregs)_CIBERSORT	0.0420	0.5688		0.0735	0.4884	0.0342	0.8707	-0.1393	0.6217	-0.1008	0.5688
T cell regulatory (Tregs)_CIBERSORT-ABS	0.0704	0.2801		0.0755	0.4708	0.0501	0.7806	-0.1427	0.6127	-0.0948	0.6021
T cell regulatory (Tregs)_QUANTISEQ	-0.0379	0.6127		0.0261	0.8121	0.1399	0.3574	-0.1302	0.6371	-0.0453	0.8121
T cell regulatory (Tregs)_XCELL	-0.0133	0.8870		-0.0551	0.6018	0.0483	0.7870	-0.0469	0.8933	-0.1507	0.3495

Spearman's Correlation Coefficient
> 0.25 / < -0.25 > 0.50 / < -0.50 > 0.75 / < -0.75

Adjust P-value
* < 0.05 ** < 0.01 *** < 0.001
